# Maintaining Quality of Genetic Counseling Services While Maximizing Efficiency: Protocol for the GC‐PRO Intervention Stepped‐Wedge Cluster Randomized Trial

**DOI:** 10.1002/jgc4.70297

**Published:** 2026-10-14

**Authors:** Deborah Cragun, Elena R. Fisher, Ashley Petersen, Heather A. Zierhut

**Affiliations:** ^1^ College of Public Health University of South Florida Tampa Florida USA; ^2^ Department of Genetics, Cell Biology, and Development University of Minnesota Minneapolis Minnesota USA; ^3^ Division of Biostatistics and Health Data Science University of Minnesota Minneapolis Minnesota USA

**Keywords:** clinical efficiency, community‐based participatory research, genetic counseling, genetic counseling research, health communication, patient‐reported outcomes, service quality

## Abstract

The rapid integration of genomic medicine into clinical care has created an unprecedented demand for genetic counseling (GC) services. Attempts to streamline care have involved integration of GC assistants, group counseling sessions, and development of educational technology supplements; meanwhile, improving the efficiency of the core clinical interaction itself remains understudied. This study will evaluate the GC‐PRO Intervention, a targeted communication training for genetic counselors designed to optimize visit time by aligning counseling with patient‐centered needs and removing processes that do not contribute to positive patient experiences. Using a stepped‐wedge cluster randomized trial design across clinical specialties (e.g., prenatal, cancer, cardiology, and general genetics) at two academic health centers, we will recruit approximately 20 genetic counselors and 994 patients. To ensure the intervention and study design are culturally responsive, we are utilizing a community‐based participatory research approach with four multicultural community advisory boards. The intervention training focuses on three evidence‐based communication skills: Tailoring (assessing and integrating patient needs and values), Streamlining (reducing time spent gathering patient medical and family history information), and Main Messaging (reducing lecturing through increasing conversational exchanges). The primary outcome is visit time, and secondary outcomes include patient‐reported measures of empowerment, activation, trust, working alliance, patient‐centered communication, and overall satisfaction. Data will be analyzed using linear and generalized mixed‐effects models to compare the intervention to standard of care, accounting for calendar time, counselor nesting, and pre‐specified patient characteristics to improve precision. We hypothesize that this intervention will significantly reduce average visit time while maintaining or improving session quality, as measured against pre‐specified non‐inferiority thresholds. With 80% power to detect a 5‐min difference in session length, this study aims to provide a scalable framework for efficient GC services without sacrificing the counselor‐patient relationship or patient‐centered outcomes.

## Abstract (highlights)

**What Is Known About This Topic:**

- The rapid expansion of genomic medicine has led to an unprecedented need for increased access to quality genetic counseling services. While alternative service delivery models have addressed logistical barriers, research is lacking on how to optimize communication within the counseling session itself to increase efficiency while maintaining quality, as defined by patient‐reported outcome and experience measures.

**What This Paper Adds to the Topic:**

- The GC‐PRO Intervention is a novel health communication approach comprising 3 skills (Tailoring, Streamlining, and Main Messaging) that focus on core counseling processes associated with positive patient outcomes while removing process items not found to contribute to quality‐ and efficiency‐focused GC encounters.
- By utilizing a stepped‐wedge trial design and community‐based participatory research, this study will test an optimization intervention to reduce visit time while ensuring that patient‐reported outcomes (e.g., trust, empowerment, activation) remain high across diverse clinical specialties and patient populations.

## Plain Language Summary

This study will test a new way for genetic counselors to focus only on what patients really want to know. By cutting out parts of the visit that may not help, we may be able to make counseling visits shorter while making sure people still feel heard and supported. The goal of this study is to make sure every patient gets high‐quality care that fits their personal values without wasting time on things that do not matter to them.

## 1 Introduction

The rapid integration of genomic medicine into standard clinical care has created an unprecedented demand for genetic services, and the infrastructure required to deliver quality genetics services is increasingly strained, resulting in challenges to access in services (Bellaiche et al. 2021; Cragun et al. 2017; Doberstein et al. 2024; Southwick et al. 2020; Villegas and Haga 2019; Wagner et al. 2024). Multiple modalities will be needed to create a more efficient infrastructure and evidence base for access to quality GC services. Incorporation of GC assistants, use of technology to streamline administrative tasks, variation in service delivery models, and supplementation with educational products have been used (Gordon et al. 2018; Greenberg et al. 2020; Hallquist et al. 2020; Pirzadeh‐Miller et al. 2017). While these structural adjustments have improved some aspects of access to GC services, they primarily focus on the logistical aspects of care coordination or patient education/consent rather than other core aspects of the clinical interaction. Less understood are the essential elements of the GC visit that are important for creating an efficient clinical practice while maintaining both counselor professional satisfaction and quality of patient care. In fact, the Institute of Medicine recognizes both patient‐centeredness and efficiency as core dimensions of healthcare quality, underscoring the importance of using clinical time effectively (Institute of Medicine (US) Committee on Quality of Health Care in America 2001).

Understanding how patient care time is spent in GC and the relationship between time spent with corresponding patient experiences and outcomes is key to defining current practice and working toward efficient, quality services. A scoping review by Glanton et al. (2025) found that studies on GC patient care time are highly variable and rarely analyze patient‐reported experiences or outcomes in relation to time spent (Glanton et al. 2025). Data suggests that shorter sessions do not inherently lead to poorer outcomes, with interventional studies showing that no significant differences in patient knowledge or satisfaction were found between standard and shorter sessions (Calzone et al. 2005; Cloutier et al. 2017). The impact on psychological outcomes may be more nuanced, with some patient subgroups demonstrating more dramatic improvements in worry with longer sessions (Goyal et al. 2002).

More research is needed to understand how the utility of visit time impacts GC session quality, thereby ensuring that time‐saving interventions do not inadvertently sacrifice patient experiences. For minoritized populations who already face systemic barriers to care, an efficient model of GC practice must be designed to maximize utility while maintaining trust and meeting patients' needs (Mittendorf, Knerr, et al. 2021; Rodriguez et al. 2023). In this way, efficiency may be defined as the optimization of GC visit time to meet each patient's informational, cultural, and linguistic needs.

The few health communication interventions in GC settings that do exist have been siloed by clinical indication, specific patient populations, or isolated session components. Studies have focused on singular tasks, such as risk communication (Edwards et al. 2008) or post‐session family genetic risk communication (Hodgson et al. 2014; Zhao et al. 2022). For example, Joseph et al. (2019) implemented a pilot intervention with low health literacy cancer patients using teach‐back and plain language (Joseph et al. 2019), and the ARIA model prioritizes relationship‐building and patient engagement by tailoring information during results disclosures (Joseph et al. 2022; Mittendorf, Kauffman, et al. 2021). Outcomes assessed in health communication interventional studies are often limited to patient comprehension and/or satisfaction, failing to capture broad indicators of quality such as patient empowerment or activation, which have been prioritized by stakeholders (Cragun and Zierhut 2018; Redlinger‐Grosse et al. 2016, 2021). Research on efficiency‐focused GC interventions should align study metrics with stakeholder‐prioritized measures of GC quality to assess outcomes that matter most to patients and providers.

In this study, we will test a GC optimization intervention training designed to align counseling with patients' desired informational needs and value assessments and eliminate counseling processes that have not been associated with positive patient outcomes based on data from GC‐PRO Stage 1. We hypothesize that modifying GC sessions based on patients' needs and preferences and removing process items found not to contribute to differences in patient outcomes will increase efficiency (i.e., reduce average visit time) and maintain or improve session quality, as measured by patient‐reported outcome measures (PROMs) and patient‐reported experience measures (PREMs). Our primary quality metrics align with stakeholder priorities, including overall perception of GC quality, patient‐centered communication, patient empowerment, patient activation, patient satisfaction, working alliance, perceived trust in GC, and informational needs met/information overload.

## 2 Methods

### 2.1 Study Overview

This study will evaluate the effectiveness of a GC optimization (e.g., efficiency and quality) intervention training using a stepped‐wedge cluster randomized trial design. Planned statistical analyses were pre‐registered on AsPredicted (#206650, submitted on 12/20/2024).

#### 2.1.1 Study Setting, Population, and Inclusion/Exclusion Criteria

This is a multi‐site study conducted at two academic health centers: the University of Minnesota‐M Health Fairview (UMN) and the University of South Florida‐Tampa General Hospital (USF). Both sites operate under the UMN single Institutional Review Board (STUDY00022971). Four clinical specialties (e.g., prenatal/reproductive, cardiology, oncology, and general genetics) will participate across the two sites.

The study will recruit to obtain approximately 994 recorded GC sessions (798 at UMN and 196 at USF) from English‐speaking adult patients or parents/legal guardians of underage patients referred for GC for a personal or family history of a prenatal/reproductive, cardiology, oncology, or general genetics indication. Patients will be excluded if they are unable to provide informed consent, cannot complete study surveys in English, or are under 18 years of age. We will include a variety of clinical indications and pre‐ and post‐test sessions.

Quota sampling will be employed to recruit an approximate racial/ethnic distribution of ~75% White non‐Hispanic patient participants and ~25% patient participants identifying as Black/African American, Hispanic/Latino, Asian, and/or other race/ethnicity per recruiting GC clinic. This quota sampling goal was chosen to balance the goals of enrolling a diverse study population while maintaining consistent racial/ethnic patient participant distributions across clusters based on patient population characteristics from GC‐PRO Stage 1.

Approximately 20 genetic counselors will be recruited to participate. Genetic counselors must be adults, Board‐certified by the American Board of Genetic Counseling, provide services in English, and counsel patients for prenatal/reproductive, cardiology, oncology, or general genetics indications. Genetic counselors who are not Board‐certified will be excluded from participation.

#### 2.1.2 Trial Design

To test an optimization intervention training, the study will use a stepped‐wedge cluster randomized trial design at the site‐specific specialty level in which clusters crossover from standard of care to the intervention at staggered time points (Figure 1). The six clusters will include four UMN specialties and two USF specialties. In this design, all genetic counselors within the same specialty at the same site will cross over from standard of care to the intervention training at the same time, greatly reducing contamination concerns. Counselors will be advised not to discuss the intervention training with those outside of their specialty. By the end of the study, all counselor participants at all sites will have received the intervention training and will counsel patients in alignment with the strategies recommended by the intervention. The order of clusters crossing over is randomized. To minimize bias, patient participants and research assistants who perform recruitment duties will be blinded to the cluster's current treatment assignment. The enrollment process will remain uniform throughout the study to minimize the risk of selection bias from systematic differences between patients who enroll after the intervention training.

**FIGURE 1:**
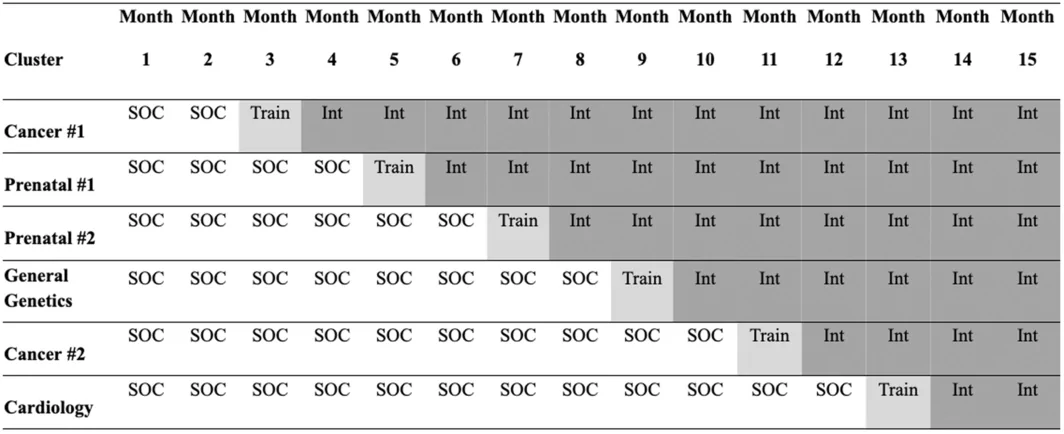
Stepped‐wedge trial design. The order in which the clusters crossover will be randomized (example shown for illustrative purposes). Int = intervention‐aligned counseling, SOC = standard of care counseling.

We will use quota sampling to recruit an average of 12 patients per month for each of the 6 clusters. The trial will have a total length of 15 months and allow each cluster a one‐month pause in recruiting during their training month, which will immediately precede the cluster's crossover to the intervention. A web‐based data dashboard (developed by A.P. using R Shiny) will be used to monitor enrollment in real time to ensure steady enrollment over the course of the trial (Chang et al. 2025). Adjustments to recruitment goals and enrollment of additional counselors will be implemented as needed to account for changes in counselor availability (e.g., medical leave, resignation).

#### 2.1.3 Primary and Secondary Outcomes

The primary outcome of the trial is visit time (min) (details of collection outlined in Section 2.4.3). Secondary outcomes will include overall GC quality, as measured by several PREMs completed post‐visit, and two PROMs completed pre‐ and post‐visit (Table 1). PREMs include: (1) overall quality of the visit, as measured by a Consumer Assessment of Healthcare Providers and Systems (CAHPS) 10‐point rating question (Hargraves et al. 2003; Liang et al. 2017; Weidmer et al. 2012); (2) patient‐centered communication, as measured by the Patient‐Centered Communication Scale (Moser et al. 2022); (3) patient information needs met/information overload, as measured by the Cancer Information Overload Scale (Jensen et al. 2014, 2017); (4) patients' perceived trust in GC, as measured by the Wake Forest Trust Scale (Hall et al. 2002); and (5) working alliance, measured by the Working Alliance Inventory (Erby et al. 2021; Hatcher and Gillaspy 2006). Two additional PREMs include the Jefferson Scale of Patient Perceptions of Physician Empathy (Kane et al. 2007) and the CollaboRATE Shared‐Decision Making (Barr et al. 2014). PROMs include (1) patient empowerment, as measured by the Genetic Counseling Outcome Scale (McAllister et al. 2011); and (2) patient activation, as measured by the Patient Activation Measure (registered trademark of Insignia Health LLC) (Hibbard et al. 2005).

**TABLE 1:** PROMs and PREMs collected from patient participants in the GC‐PRO Intervention study.

Measure	Number of items	Response scale	Sample item
*PROMs (measured pre‐ and post‐visit)*			
Patient Activation Measure (a registered trademark of Insignia Health LLC)	10	5‐point Likert scale	I am confident that I can follow through on the plan when I get home.
Genetic Counseling Outcome Scale	17	5‐point Likert scale	I know how to get the non‐medical help I/my family needs (e.g., educational, financial, social support).
*PREMs (measured post‐visit)*			
CAHPS 10‐point quality rating	1	11‐point scale (0 to 10)	Using any number from 0 to 10, where 0 is the worst genetic counseling possible and 10 is the best genetic counseling possible, what number would you use to rate the genetic counseling visit?
Patient‐Centered Communication Scale	7	4‐point scale	How often did the genetic counselor help you deal with feelings of uncertainty about your health or health care?
Cancer Information Overload Scale	5	5‐point Likert scale	What we talked about in the visit left me feeling overloaded.
Wake Forest Trust Scale	10	5‐point Likert scale	My genetic counselor is totally honest in telling me about all of the different options available to me.
Working Alliance Inventory	10	7‐point Likert scale	I feel the genetic counselor cares about me even when I do things that he/she does not approve of.
CollaboRATE Shared Decision Making	3	11‐point scale (0 to 10)	How much effort did the genetic counselor make to listen to the things that matter most to you about the health condition?
Jefferson Scale of Patient Perceptions of Physician Empathy	5	7‐point Likert scale	My genetic counselor understands my emotions, feelings and concerns.

*Note:* Patient‐reported outcome and experience measures collected from the patient participants through quantitative surveys, including the number of items, response scale for each measure, item examples, and timing of measure collection.

#### 2.1.4 Community‐Based Participatory Research (CBPR) Approach

This study will incorporate a CBPR approach using 4 multicultural community advisory boards (CABs) and research team members who are also members of understudied communities in the U.S., including Black/African American, Latina, Somali, and Hmong communities (Fisher et al. 2024). The CABs will meet twice per year to provide input on the intervention, findings, and dissemination plans.

### 2.2 GC Optimization Intervention

Skill development and selection were based on prior health communication literature, as well as on counseling preferences and issues uncovered through patient interviews during GC‐PRO Stage 1. The three most important skills were selected for inclusion in the intervention (Table 2). During the intervention development process, the skill concepts and descriptions were presented to the CABs for review; feedback was used to make modifications as part of an iterative process. Below, each skill is described along with its rationale and evidence base as well as summaries of CAB feedback.

**TABLE 2:** GC‐PRO Intervention skill descriptions and timing of implementation within the session.

Skill	Skill description and components	Timing of implementation
Skill 1: Tailoring	*Assess*: Provide context then ask direct, open‐ended questions to get to know the patient *Acknowledge*: Verbally acknowledge or clarify the patient's responses *Apply*: Integrate answers into the session	Beginning of session: Get to know patient Assess and acknowledge Throughout the session: Apply their answers
Skill 2: Streamlining	*Explain why*: Explain why or how the information you're collecting is being used in the session *Streamline*: a. Ask only pertinent questions b. IF NEEDED: Redirect patients with “Yes, AND”	During information‐gathering components, such as: Medical history intake Family history intake Other risk assessment sections
Skill 3: Main Messaging	*For each main message*: *Ask*: Assess prior knowledge or thoughts with an open‐ended question *Tell*: Give brief amounts of information (1 main message at a time) *Ask*: Elicit what they think about the main message and determine whether more details are desired BEFORE moving onto the next main message	2 key information‐giving areas: Educating Action planning (toward the end of the session)

*Note:* Descriptive summaries of the GC‐PRO intervention skills (e.g., Tailoring, Streamlining, and Main Messaging) along with recommended timing of implementation within the session.

#### 2.2.1 Skill 1: Tailoring

Tailoring refers to incorporating a patient's values, beliefs, experiences, and priorities into a GC session by adapting the content, focus, and delivery of information to align with what matters most to the patient. For this study, the Tailoring process involves assessing and acknowledging the patient's interests and experiences and noting how this adjusts the type or amount of information provided. Tailoring is conceptualized as a three‐step process: (1) Assessment, (2) Acknowledgement, and (3) Application. Tailoring begins by establishing the context of the visit (i.e., the reason for referral) and then asking two or more direct, open‐ended or “what else” questions related to the patient's most important informational needs, values, or goals relevant to the session. These questions should occur near the beginning of the session. Assessment questions may also include brief personal inquiries that help counselors better understand the patient's perspective.

The second step involves verbally acknowledging or clarifying the patient's responses to the assessment questions at least two times. These responses should occur shortly after the patient reveals their needs/concerns/priorities and may include reflective statements, exploratory comments, or follow‐up questions. This step demonstrates active listening and ensures the counselor accurately understands the patient's concerns and priorities.

The final step involves incorporating the patient's concerns, values, or informational needs at least twice during the session. This may include linking explanations, recommendations, or counseling content back to the patient's expressed concerns or needs. Integrating patient‐identified priorities or personal information allows counselors to adapt content in ways that are directly relevant to the patient.

Tailoring communication supports patient‐centered care by demonstrating attentive listening and ensuring that clinical discussions address the patient's priorities. Research suggests that patients prefer when genetic counselors assess their informational needs and concerns at the start of an encounter (Redlinger‐Grosse et al. 2021). Similarly, using direct open‐ended questions and follow‐up “what else” prompts has been shown to more effectively elicit patient agendas (Allgood et al. 2023). In addition, findings from GC‐PRO Stage 1 indicate that patients rated counseling sessions higher when they perceived them as individualized and responsive to their priorities (Peifer et al. 2026).

CABs firmly supported the tenet of Tailoring and recommended encouraging counselors to first provide context regarding the content and expectations of the GC visit. They expressed that it would then enable patients to understand which values, beliefs, or concerns are relevant to the appointment. CABs also suggested that counselors “get to know individuals first” through genuine relationship‐building questions, which could allow for further personalization.

#### 2.2.2 Skill 2: Streamlining Information Gathering

Streamlining has two components: (1) Explain Why and (2) Streamline. Explain Why involves the counselor explaining how they are planning to use the information gathered from the patient or why a group of related questions is being asked. This skill should be used at least once, but can be used more than once during any intake portions of the session.

The second component of the skill has two parts: (2a) Only ask patients questions that are critical to assessing risks or planning patient care, and (2b) Redirect conversations using “Yes, AND” or another strategy to focus talkative patients on more important or relevant topics if they veer off on tangents. When conversations veer from important topics and/or are not serving a professional purpose, redirection strategies may be used at the counselors' discretion. “Yes, AND” is one redirection strategy defined as first acknowledging the patient's statement, saying “and” immediately, then following up with either a question about what the counselor wishes to address next and/or a suggestion of how to address the patient's stated need.

Streamlining information gathering supports efficient, patient‐centered GC by ensuring that questions are purposeful, transparent, and directly relevant to decision‐making and management plans. Explaining why questions are being asked helps patients understand the relevance of the information they provide and clarifies how it will be used. Prioritizing only the most clinically relevant medical and family history questions helps prevent patients from feeling “bombarded” with information requests and allows more time for education, shared decision‐making, and action planning.

Findings from GC‐PRO Stage 1 indicate that the time spent gathering medical and family history information was one of the primary factors contributing to longer session duration (Glanton et al. 2025) and a factor in lower overall PREMs. Furthermore, some GC‐PRO participants reported frustration when counselors asked numerous medical or family history questions that were not subsequently used during the visit (Peifer et al. 2026). Similarly, standardized patients participating in simulated GC encounters rated sessions more positively when the GC refrained from extensive information gathering (Cragun et al. 2025). Acknowledgement followed by redirection strategies, such as the “Yes, and” approach, supports both relational communication and efficient use of limited session time. Communication research suggests that acknowledging a patient's perspective before guiding the conversation toward another topic can reduce defensiveness and support collaborative dialog (Nguyen et al. 2024; Resnicow and McMaster 2012).

There was strong agreement across all CABs regarding the importance of providing patients with more context for why they are asked various medical and family history questions. CABs noted that rapport and trust may be negatively impacted when providers do not acknowledge how the information is being used, particularly when patients spend significant time completing questionnaires or when the information is considered sensitive.

#### 2.2.3 Skill 3: Main Messaging in Education and Action Planning

Main Messaging involves repetition of a three‐step process (“Ask‐Tell‐Ask”) when educating (discussing testing options, medical management, and test results) and during action planning (collaboratively developing a plan which outlines actions the patient can take based on their risk assessment, decision(s) made, test results, or management guidelines). To implement Main Messaging, counselors identify 3 to 5 main messages (i.e., key information that may impact decision‐making or action‐taking after the visit) relevant to the patient's case and then implement the Ask‐Tell‐Ask process for *each* main message in education and/or action planning. Main Messaging requires that educational elements not directly related to the patient's situation should be eliminated.

Ask‐Tell‐Ask involves asking about the patient's existing knowledge about a key topic, providing targeted information, and then asking follow‐up questions to assess understanding, reactions, or informational needs. Our version of Ask‐Tell‐Ask was modified from motivational interviewing (MI), in which the initial Ask is designed to obtain permission to discuss a specific topic (Ash 2017; Baldry et al. 2022). The reason for this modification was that genetic counselors are typically obligated to cover certain topics (Brown et al. 2018; Zale et al. 2022); thus, asking and then ignoring the patients' wishes would be insensitive. Therefore, in our process, the first Ask is defined as an assessment of the patient's prior knowledge or thoughts on a topic and is conducted using an open‐ended question, or a closed‐ended question or statement that elicits an open‐ended response from the patient (i.e., more than a “yes” or “no” answer). The first Ask should be implemented *before* giving a main message in order to determine what the patient already knows and/or the topics most salient to the patient. The Tell is defined as providing a concise (e.g., within 2–3 sentences) summary of a main message without the use of jargon or medical terminology (unless it is necessary and defined). Finally, the second Ask involves eliciting the patient's thoughts on the main message or determining whether more details are desired. The second Ask should be implemented through either an open‐ended question or a closed‐ended question, statement, or pause that elicits an open‐ended response or question from the patient. Counselors are encouraged to include specific questions that relate directly to the main message to avoid a scripted feel and maximize patient responsiveness.

After Ask‐Tell‐Ask has been implemented for one main message, counselors will move to the next main message and apply Ask‐Tell‐Ask again for that message. This process is not intended to be formulaic, but rather to create a back‐and‐forth dialog in which the patient is actively participating and the key messages are intentionally broken up. This cyclical approach encourages collaborative communication and ensures that information provided is conversational, relevant, understandable, and responsive to patient priorities.

A key rationale for Main Messaging is that patients and patient advocates report preferring conversational exchanges (rather than extended monologues) that avoid overly technical, highly detailed, or unrelated information (Redlinger‐Grosse et al. 2021). Ask‐Tell‐Ask also allows clinicians to adapt information delivery to individual patients' levels of interest and informational needs. Patients vary in how much detail they want during GC sessions and may only want brief information about some options (Hurley et al. 2012). Excessive jargon or unsolicited technical explanations can contribute to information overload, confusion, and disengagement, particularly when numerical or risk information is presented without context (Joseph et al. 2017, 2019). Finally, providing information that is clearly connected to clinical purpose and next steps can improve patients' ability to act on the information they receive. Patients consistently report valuing clear guidance about actionable next steps and appreciate opportunities to ask questions and discuss potential plans, which in turn is associated with a greater likelihood of acting on health information (Winchester et al. 2022; Zierhut et al. 2016). Additionally, prior work has found that when standardized GC patients rated sessions highly, they often tied the value of the information to how it helped activate or empower them (Cragun et al. 2025). In contrast, comments on the value of information from lower‐rated sessions occurred in conjunction with comments about too much information and jargon (Cragun et al. 2025).

Main Messaging was strongly supported by all CABs. CABs acknowledged that there is a balance in providing information: some patients do want details, while others do not. CABs recommended that counselors get to know the person first (e.g., Tailoring—Assessment) to understand the level of detail each person needs. Lastly, CABs acknowledged that some patients will not know what they need or what questions to ask. Therefore, it was suggested that counselors provide context and ask open‐ended questions to elucidate how best to meet patients' informational needs.

### 2.3 Study Procedures

#### 2.3.1 Genetic Counselor Participants

Genetic counselors who meet all inclusion criteria will be recruited through group presentations and individual outreach. Interested counselors will review and sign an electronic informed consent form and complete a baseline survey (Section 2.4.1).

Enrolled genetic counselors will complete the following: (1) conduction of standard‐of‐care GC followed by crossover to the intervention training at staggered timepoints, (2) intervention training at the crossover point, (3) fidelity assessment at two timepoints (2 months and 4–6 months) after the intervention training was received, (4) surveys assessing attitudes about the intervention training during each fidelity assessment and after completion of the trial, and (5) optional semi‐structured interview at the 4–6 months post‐training timepoint to further assess opinions about the intervention skills and discuss the extent to which they are utilizing the skills (i.e., perceived fidelity to the intervention).

Research incentives (e.g., prepaid debit cards) will be provided to participating genetic counselors for each training activity and fidelity assessment activity. Counselors will be compensated $75/h for their time and may earn up to $1100 total in incentives for their participation.

##### 2.3.1.1 Intervention Training and Review of Fidelity Assessments

During the intervention training month, no patient participants will be recruited in the cluster. Genetic counselors will be notified of their cluster's randomization to the intervention training approximately 1 month in advance. The month‐long training will include: (1) self‐paced learning about the skills and completion of short assignments (2–3 h); (2) virtual group discussion, reflection, and practice (2 h); (3) completion of an hour‐long mock session with a standardized patient, followed by review of written feedback (1.5 h), and (4) completion of a second mock session with corresponding review of feedback (1.5 h). In total, the training is anticipated to take 8 h.

Initial self‐paced learning in Week 1 will include written PowerPoint materials and online training videos for each skill (see Supporting Information). Counselors will complete short reflections or “skills checks” to brainstorm ways to integrate the skills into their clinical practice. Group discussion with the research leaders during Week 2 will involve clarification of skill details, review of questions that arose from skills checks, and short role plays. A one‐page reminder of key skill criteria will be provided. Mock sessions in Weeks 3 and 4 will be based upon a specialty‐specific, common clinical scenario modified from Cragun et al. (2025). Patient actors (e.g., research assistants at UMN and USF) will be provided with a detailed patient scenario to standardize patient actors' thoughts, feelings, and behaviors across sessions. Sessions will be recorded and reviewed by H.A.Z. and D.C. for fidelity to the intervention skills. Feedback will be provided via annotation of the recorded video and completion of the Fidelity Data Review Form with suggestions for improvement. Participating counselors will review the feedback and complete a short attestation survey. Counselors will have the opportunity to complete a third mock session and/or individualized one‐on‐one training with the research leaders if they have low fidelity to the skills in the first two mock sessions.

Two months following the training month, genetic counselors will receive an additional “tips and tricks” sheet regarding skill implementation. At that time, counselors will also receive completed Fidelity Data Review Forms with comments for two randomly selected cases. Counselors with low fidelity to the skills at this time point will be asked to meet with a research leader to discuss barriers to skill implementation. After reviewing the fidelity data, genetic counselors will complete post‐training fidelity reflection surveys (Section 2.4.1).

At 4‐ to 6‐month post‐intervention training, counselors will receive a summary of their “skill implementation scores” along with the study‐wide average fidelity scores of other genetic counselor participants who have also completed the training. Each counselor's skill implementation scores will be based upon 2 randomly selected cases from each of the past 4 to 6 months (i.e., a total of 8–12 cases). After reviewing their fidelity scores, counselors will complete their second post‐training fidelity reflection survey (Section 2.4.1).

#### 2.3.2 Patient Participants

Patient participants will be recruited to complete: (1) a pre‐visit survey, (2) an audio recording of their GC session, and (3) a post‐visit survey. Recruitment will occur via a multi‐pronged approach using phone call outreach, mailed letters, electronic health portal messages, and/or in‐person recruitment before their GC appointment. This multi‐pronged recruitment approach, informed by the study's CABs, has shown success in recruiting a diverse participant sample during GC‐PRO Stage 1 (Fisher et al. 2024). Patients who complete the pre‐ and post‐visit surveys and the session recording will be emailed a $30 electronic gift card.

### 2.4 Measures

#### 2.4.1 Genetic Counselor Participant Measures

Demographic information (e.g., age, gender identity, race/ethnicity, years of experience, and training program), Genetic Counseling Self‐Efficacy Scale (Caldwell et al. 2018), the Interpersonal Reactivity Index (Davis 1983), and a novel scale assessing the importance of 5 GC session components (counseling, decision‐making, history taking, teaching, and testing logistics) will be collected in the baseline survey (Table 3).

**TABLE 3:** Measures collected from genetic counselor participants in the GC‐PRO Intervention.

Measure	Number of items	Response scale	Sample item	Timing of collection
Genetic Counseling Self‐Efficacy Scale	38	7‐point Likert scale	Please rate how certain you are that you can perform this competency today in a genetic counseling session: build trust between myself and the client	Trial initiation (month 0) Trial completion (after month 15)
Interpersonal Reactivity Index	14	5‐point Likert scale	I often have tender, concerned feelings for people less fortunate than me	Trial initiation (month 0) Trial completion (after month 15)
Acceptability of Intervention Measure	4	5‐point Likert scale	I like the GC‐PRO Intervention Skills	2 months post‐training 4–6 months post‐training Trial completion (after month 15)
Intervention Appropriateness Measure	4	5‐point Likert scale	The GC‐PRO Intervention Skills seem applicable	2 months post‐training 4–6 months post‐training Trial completion (after month 15)
Feasibility of Intervention Measure	4	5‐point Likert scale	The GC‐PRO Intervention Skills seem doable	2 months post‐training 4–6 months post‐training Trial completion (after month 15)
Importance of GC Session Components	5	7‐point Likert scale	Please rate the importance of each of the components below within a genetic counseling session: History taking/risk assessment components	Trial initiation (month 0) Trial completion (after month 15)

The 2‐month and 4–6 month post‐training fidelity reflection and review surveys will contain the Acceptability of Intervention Measure (AIM), Intervention Appropriateness Measure (IAM), and Feasibility of Intervention Measure (FIM) scales (Weiner et al. 2017), as well as open‐ended questions regarding skill implementation in practice (Table 3).

Lastly, a final survey administered at the conclusion of the trial will again assess the Genetic Counseling Self‐Efficacy Scale, Interpersonal Reactivity Index, the Importance of GC Session Components scale, and the AIM, IAM, and FIM scales.

#### 2.4.2 Intervention Fidelity Assessment Measure

An iterative process was used to develop checklist criteria to assess fidelity to the intervention within each audio‐recorded GC session. A Fidelity Data Review Form was developed with the following characteristics: (1) clear instructions to assess skill completion; (2) succinct, dichotomous or frequency‐based response options; (3) option to determine if opportunities for skill implementation were present but not acted upon; (4) ability to capture usage across specialties; and (5) completion of form in real time. In total, the final Fidelity Data Review Form contains 17 items with variable point values (4 items addressing Tailoring, 3 items addressing Streamlining, and 10 items addressing Main Messaging). Additionally, time spent on information‐gathering and information‐giving portions of the session will be documented to measure the impact of Streamlining and Main Messaging, respectively.

Research assistants will be trained to code audio‐recorded sessions using the Fidelity Data Review Form through an iterative process involving group discussion, individual practice, and comparison with research leader‐coded sessions. Coders will be blinded to whether the session is during the standard‐of‐care or intervention periods. We will continue to assess intercoder reliability throughout the study.

#### 2.4.3 Patient Participant Measures

The pre‐visit survey will collect: demographic information (including age, gender identity, race/ethnicity, education level, and income); awareness of GC; history of prior genetic testing; baseline health literacy (Chew et al. 2008); Genetic Counseling Outcome Scale (Grant et al. 2019; McAllister et al. 2011); and the Patient Activation Measure (a registered trademark of Insignia Health LLC) (Hibbard et al. 2005) (Table 1).

The post‐visit survey will collect: Information Overload; Genetic Counseling Outcome Scale; Patient Activation Measure (a registered trademark of Insignia Health LLC); Patient‐Centered Communication Scale; Working Alliance Inventory; CollaboRATE Shared‐Decision Making (Barr et al. 2014); Jefferson Scale of Patient Perceptions of Physician Empathy (Kane et al. 2007); and a series of questions based upon the CAHPS Clinician & Group Survey Supplemental Items (Table 1). In addition to the Wake Forest Trust Scale, an adapted measure examining trust in the genetic counselor was developed based on various measures of medical trust/mistrust in clinicians (Dugan et al. 2005; Merenstein et al. 2023). The inclusion of a trust measure was directly informed by this study's CABs.

#### 2.4.4 Other Session Characteristics

Visit time (the primary outcome) is defined as the total time spent in the GC encounter, from the beginning of the session until the conclusion of all counseling. Visit time is measured as the total length of the de‐identified GC audio recording, which involves removal of protected health information of the patient and genetic counselor participants, silent space in the recording when a patient or counselor exits the room, discussion of the GC‐PRO research study, and adjacent consultations with other providers (such as maternal‐fetal medicine physicians). De‐identified session length will be measured in a standardized, blinded manner.

### 2.5 Statistical Power and Planned Analyses

#### 2.5.1 Statistical Analysis Plan

##### 2.5.1.1 Genetic Counselor Participant Analyses

Survey responses from counselor participants will be summarized using descriptive statistics, including frequencies/percentages and means. We will characterize trends in participants' perceptions of the intervention using the AIM, IAM, and FIM measures at 2‐months post‐training, 4–6 months post‐training, and at the conclusion of the trial. Interviews from genetic counselor participants will be transcribed, de‐identified, and analyzed via qualitative analysis to complement the quantitative survey data.

We will assess for any pre‐post changes in self‐efficacy via the Genetic Counselor Self‐Efficacy Scale. Additionally, although we do not expect any changes in counselor empathy from trial initiation to completion, we will administer the Interpersonal Reactivity Index at the conclusion of the trial to ensure that empathy did not significantly change.

###### 2.5.1.1.1 Fidelity

Interrater reliability will be assessed using Cohen's kappa and Krippendorff's alpha. We plan to code two cases per genetic counselor per month during the intervention phase, and one case per counselor in the two standard‐of‐care months preceding the training month to estimate the prevalence of the intervention skills in the GC's standard practice. For each coded session, we will calculate an overall score that represents the use of the intervention skills in the session. The score will include 6 possible points for each skill (Tailoring, Streamlining, and Main Messaging) for a total of 18 points, with higher scores indicating a more complete use of the intervention skills.

We will also calculate the proportion of time spent information‐gathering and information‐giving in standard‐of‐care vs. intervention phases to determine if information‐gathering time and information‐giving time decreases.

##### 2.5.1.2 Patient Participant Analyses

Demographic information will be summarized using descriptive statistics to characterize patient participants. To estimate the effect of the intervention training vs. standard GC practice on the primary outcome of visit time, we will fit a linear mixed‐effects model using patient‐level data, accounting for the nested structure of patients within genetic counselors within site‐specific specialties. We will use fixed effect predictors for randomized treatment (intervention vs. standard of care), time period (months 1–15), type of appointment (specialty‐specific pre‐ or post‐test), and site‐specific specialty (prenatal, cancer, cardiology, and general genetics across the two sites) and nested random effects for counselor and time period to account for the correlation of outcomes from patients who were counseled by the same counselor. Given the stepped‐wedge design, more patients will receive the intervention (vs. standard of care) as time passes. Thus, we will adjust for the potential confounder of calendar time, which can control for other changes in the way that care is delivered over time that may impact visit time. We will use an intent‐to‐treat analysis in which all genetic counselors are included regardless of their intervention fidelity. We will perform a secondary analysis that additionally includes time from intervention training as an effect modifier (i.e., an interaction between treatment and time from intervention training) in the model in order to investigate whether the intervention training has a larger impact as the genetic counselors become more comfortable and have more practice implementing it with patients.

The secondary outcomes will be analyzed in a similar manner using linear or generalized mixed‐effect models, as appropriate. To assess whether the PROMs/PREMs were maintained at an acceptable level after the intervention training, we will compare the lower (or upper, depending on the scale direction) bounds of the 95% confidence intervals for the estimated means to pre‐specified acceptable levels for patient care, which were chosen based on clinical judgment and data from GC‐PRO Stage 1. The threshold scores are: 9 out of 10 (patient perception of overall GC quality, CAHPS); 3 out of 4 (Patient‐Centered Communication Scale); 2 out of 5 (Information Overload Scale); 3.5 out of 5 (Wake Forest Trust Scale); 5 out of 7 (Working Alliance Inventory). Additionally, we will use the pre‐intervention average (allowing for a non‐inferiority margin) for the Genetic Counseling Outcome Scale and the Patient Activation Measure (registered trademark of Insignia Health LLC).

We plan to perform a complete case analysis in which those patient participants who enroll and complete the pre‐survey but fail to complete the post‐survey are excluded from analyses requiring the post‐survey data. Given the post‐survey is required to receive the incentive, there is expected to be minimal missingness. Additionally, the primary outcome of visit time can be ascertained without post‐survey completion, and thus, no missing data is expected. If there is a non‐negligible amount of missing data, a sensitivity analysis using multiple imputation with multivariate imputation by chained equations will be performed. Statistical analyses will be performed using R Statistical Software (version 4.5.3 or later; R Core Team 2021).

#### 2.5.2 Power Calculations

With a total sample size of *n* = 994 successfully recorded GC sessions, we will have 80% power at the 0.05 level to detect a mean difference of 5 min in visit length between sessions conducted via standard of care vs. post‐intervention training. The patient participants will be enrolled across the six clusters, which have varying expected numbers of genetic counselor participants and monthly patient participant enrollment. We expect to enroll a total of 20 counselors: 5 in cluster 1 (4 patient participants/counselor/month), 4 in cluster 2 (4 participants/counselor/month), 2 in cluster 3 (3 participants/counselor/month), 5 in cluster 4 (3 participants/counselor/month), 2 in cluster 5 (3 participants/counselor/month), and 2 in cluster 6 (4 patients/counselor/month). If the number of counselors differs from our projection, an updated power calculation will be performed to adjust the monthly recruitment targets to ensure we maintain 80% power. Based on the data collected in GC‐PRO Stage 1, we expect the residual standard deviation of visit length to be approximately 11 min, a within‐period intra‐cluster correlation of 0.3, and a cluster auto‐correlation of 0.6. The statistical power depends on the order in which the clusters cross over, since the clusters have varying numbers of counselors and expected monthly recruitment. The reported power of 80% is the average power over all possible cluster orderings. The minimum and maximum power are 77% and 82%, which correspond to the randomized order being the least or most favorable (in terms of statistical power). Power calculations were performed using the swCRTdesign R package (Hughes et al. 2024).

## 3 Conclusion

This study addresses an important gap in the GC literature by focusing on modifiable communication skills that have the potential to improve efficiency and quality of the counseling interaction. By utilizing Tailoring, Streamlining, and Main Messaging, counselors may focus visit time on the elements most relevant to patients' needs. To ensure the proposed intervention addresses processes that are important to patients and does not inadvertently widen health disparities, the intervention was developed using a CBPR approach. If successful, this intervention may provide an evidence‐based framework for improving the efficiency of GC services while maintaining patient‐centered care. Such approaches may be particularly important as demand for genomic services continues to expand and health systems seek scalable evidence‐based strategies to increase access without compromising quality.

## Author Contributions

**Deborah Cragun:** conceptualization, funding acquisition, writing – original draft, writing – review and editing, methodology. **Elena R. Fisher:** conceptualization, writing – original draft, writing – review and editing, methodology, project administration. **Ashley Petersen:** conceptualization, writing – original draft, writing – review and editing, methodology. **Heather A. Zierhut:** conceptualization, funding acquisition, writing – original draft, writing – review and editing, methodology, supervision.

## Funding

This work was supported by the National Human Genome Research Institute (R01HG011916) and the National Center for Advancing Translational Sciences (UL1TR002494).

## Disclosure

AI Statement: No artificial intelligence tools were used to develop this manuscript.

## Ethics Statement

Approval for this study was obtained from the University of Minnesota single Institutional Review Board on September 13, 2024 (STUDY00022971). Informed consent will be obtained from all participants prior to enrollment.

## Conflicts of Interest

The authors declare no conflicts of interest.

## Supporting information


**Data S1:** Genetic Counseling Processes Result in Outcomes

## Data Availability

Data sharing not applicable to this article as no datasets were generated during the current study. The GC‐PRO Intervention training materials are publicly available in the Supporting Information of this article.
