# Supplementary material for: Maintaining Quality of Genetic Counseling Services While Maximizing Efficiency: Protocol for the GC‐PRO Intervention Stepped‐Wedge Cluster Randomized Trial
Source: J Genet Couns. 2026 Oct 14;35(5):e70297. doi: 10.1002/jgc4.70297 (PMC13662952; doi:10.1002/jgc4.70297)
Supplement: Supplementary file 1 [file JGC4-35-0-s001.pdf]

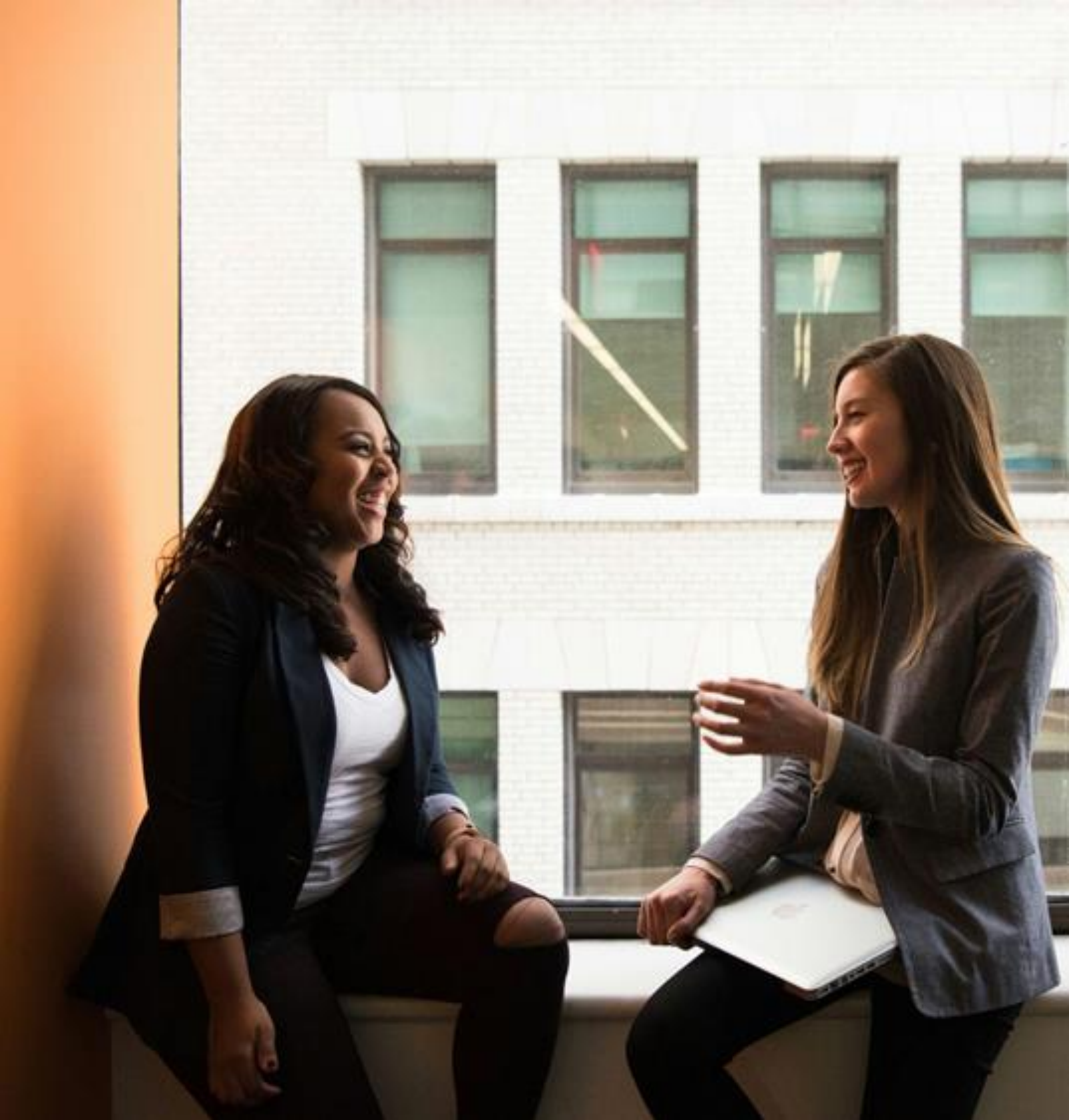

Genetic Counseling Processes Result in Outcomes:

Tailoring

Skill #1

Week 1

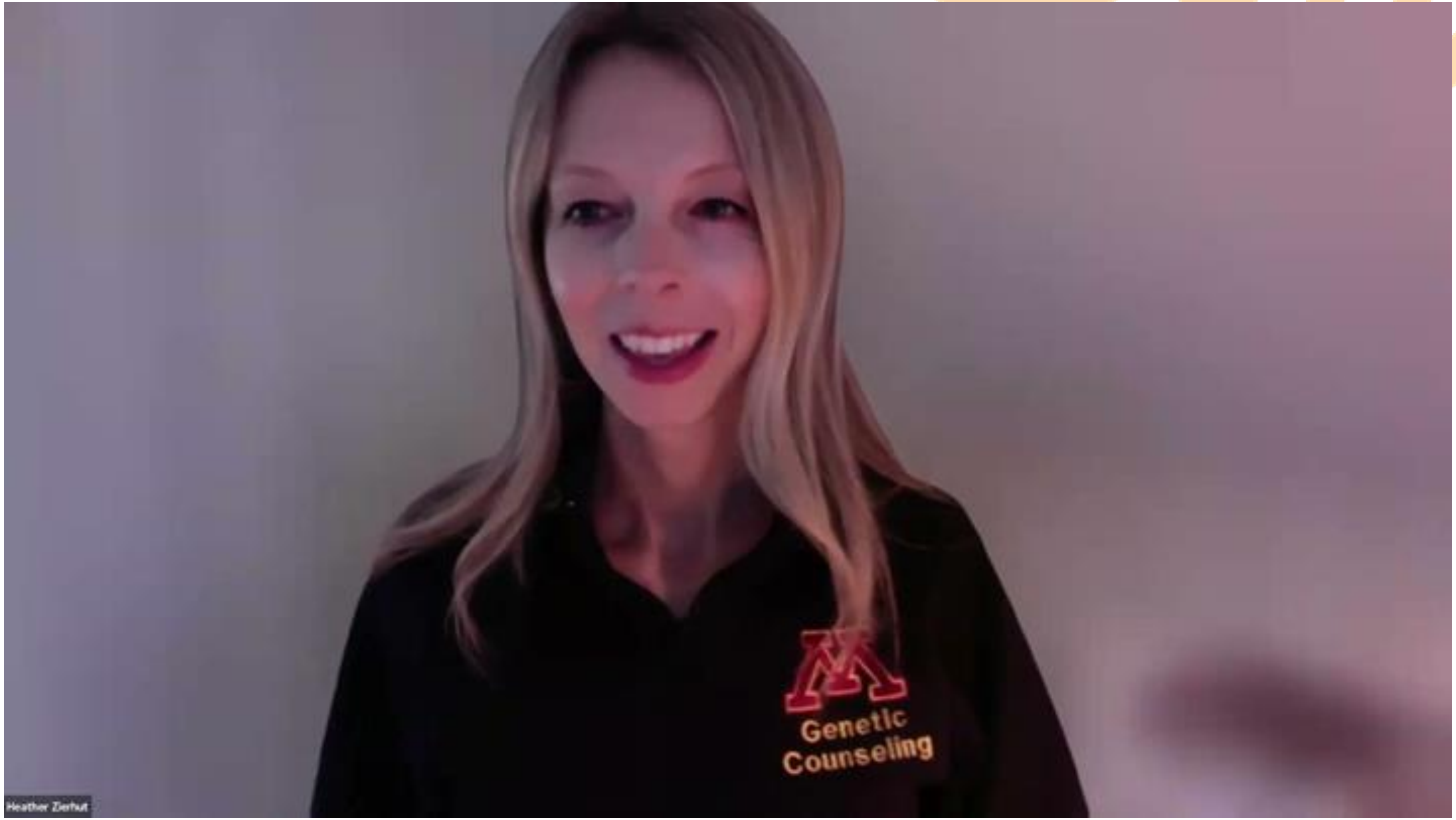

Watch this video to review content together,
or move through the slides at your own pace!

GC-PRO

Confidentiality reminder:

Please do not share the content of any GC-PRO training materials with others, including other participating GC-PRO genetic counselors at your institution. Thank you!

GC-PRO

Outline

1. Why tailoring matters
2. What’s the skill and how to use it
3. Decide how you will use “taylor-swifting” in your sessions

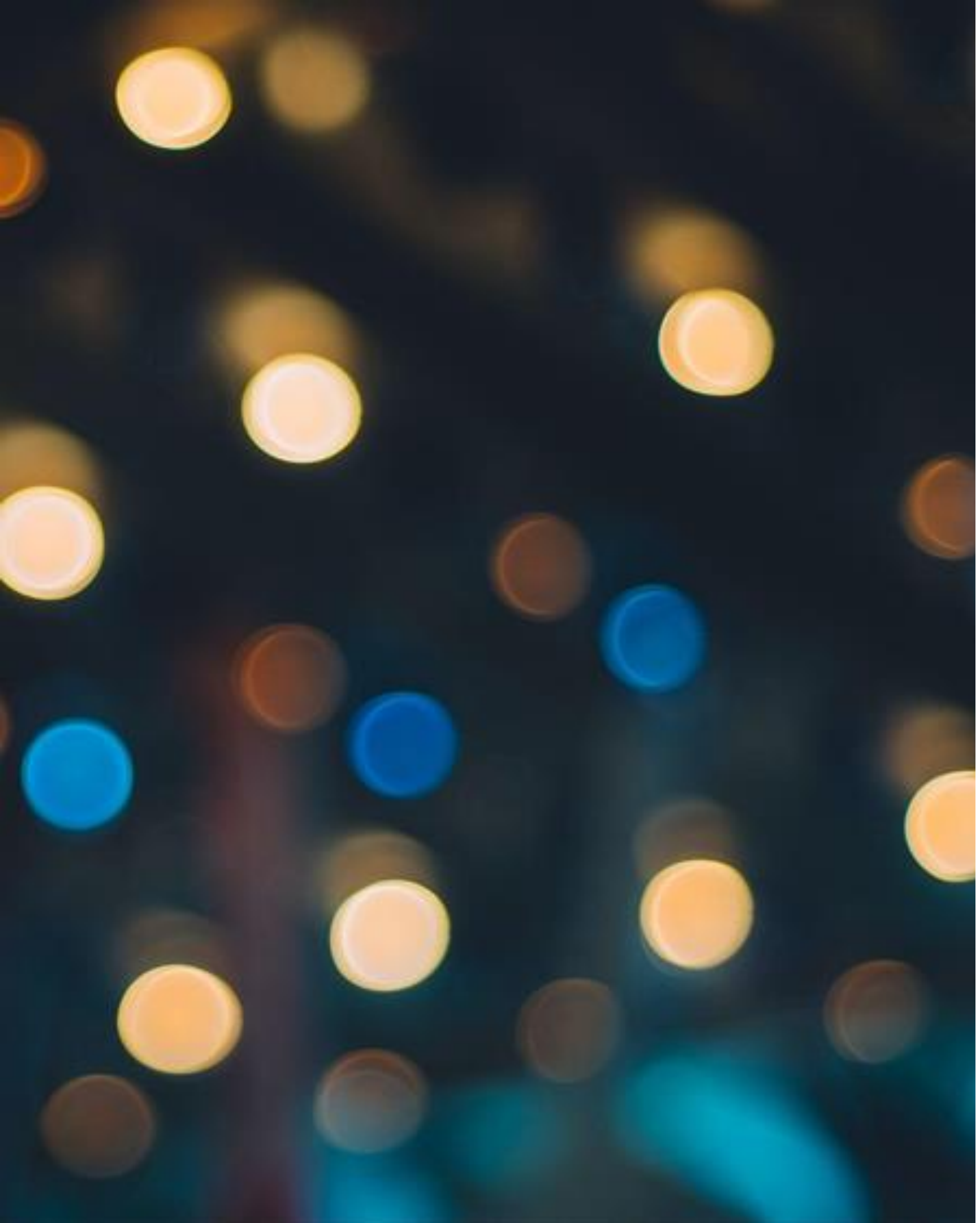

Rationale for use and evidence behind the skill

Tailoring Overview

- Tailoring can take many forms:
  - Infusing a person's **values, beliefs,** or what's most important to them within a session
  - **Acknowledging** a person's experiences or interests throughout
  - **Changing** the amount or types of information provided

- Tailoring requires you to first ***assess*** patients' needs
  - Ask questions to **understand** what's most important to the patient
  - ***Elicit*** the patient's agenda, concerns, values, etc.

GC-PRO

Why tailoring?

Shows **listening** & helps you **meet their needs**

- Patient advocates want their **informational needs assessed** (Redlinger-Grosse et al., 2021)
- Standardized patients **appreciated tailoring** during mock sessions with real counselors *(manuscript in press at JoGC)*
- From GC-PRO stage 1, patients **rated sessions highly** that were **more personalized** *(manuscript under review at JoGC)*

GC-PRO

Why tailoring?

Helps to **elicit** what patients **value** most

Information

"Like, **I don't need to talk about my feelings always**… I mean like, it matters, but it doesn't matter because **what we really needed was the information**."

- *GC-PRO Participant Interview*

Feeling heard and supported

"I always care about providers who feel **caring** and understanding and **empathetic**. And that's the feeling I got… a lot of times **that's more important to me even than the information**."

- *GC-PRO Participant Interview*

GC-PRO

Evidence behind direct, open-ended questions

- Using these types of questions has been shown to **better meet the needs** of patient's agenda

- **One word** can make a big difference in **eliciting agendas:**
  - **Direct** questions and "**What** else" questions are **BETTER*** than "**Anything** else" questions or **Personal** State (e.g. How've you been?) questions

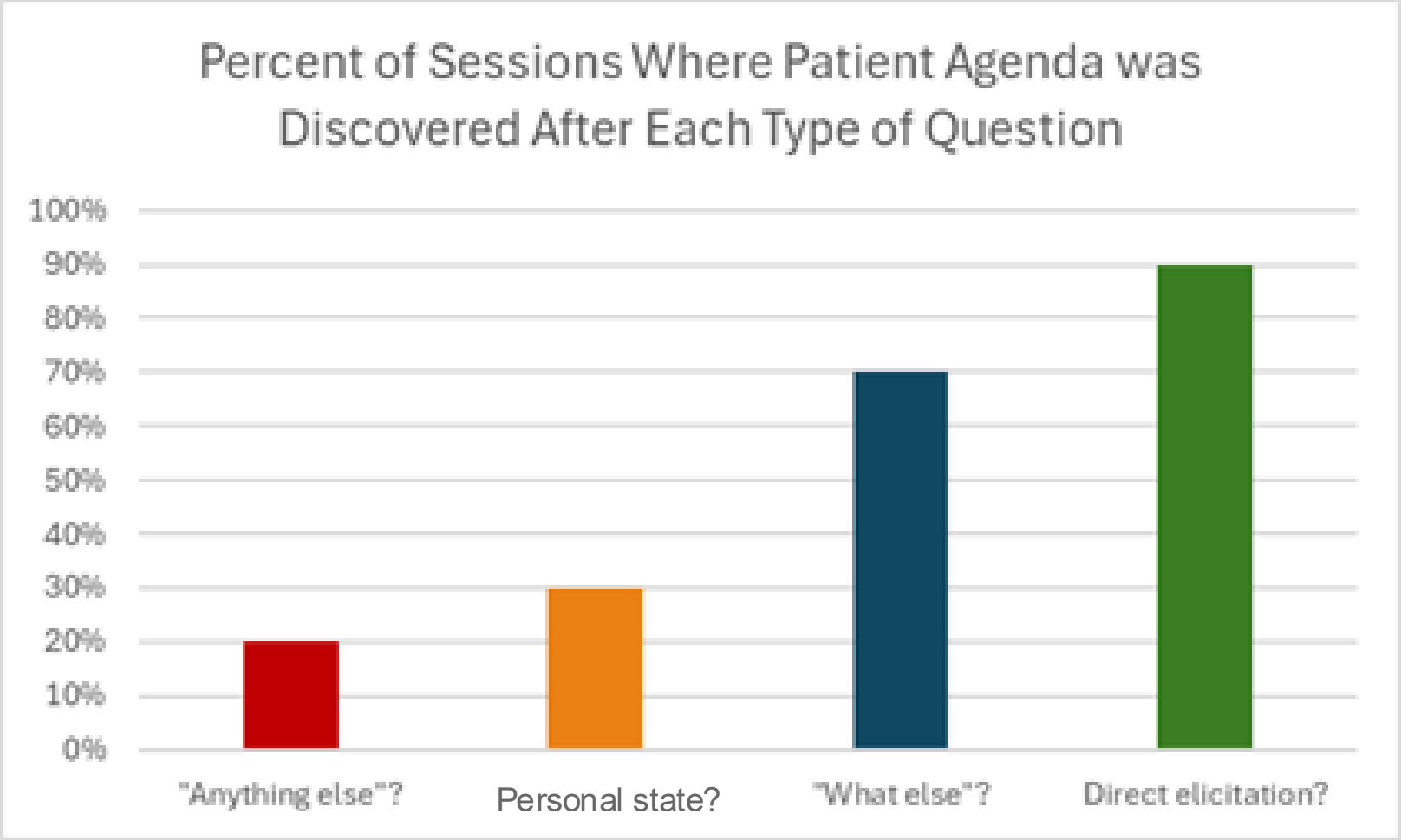

[Allgood et. al, 2023]

***BUT ONLY** if you wait for a response

GC-PRO

Why tailoring?

Enhances **conversational** flow and **personalization** of sessions

- Following a predetermined agenda and *not* personalizing sessions is **noticed**

"I felt rushed… **pressured** to follow their [GC's] format." - *Standardized patient in lower-rated session*

- Patients appreciate conversational exchange of information

"Having the face-to-face, **back and forth dialogue** also helps make you feel **more comfortable**" - *Standardized patient in higher-rated session*

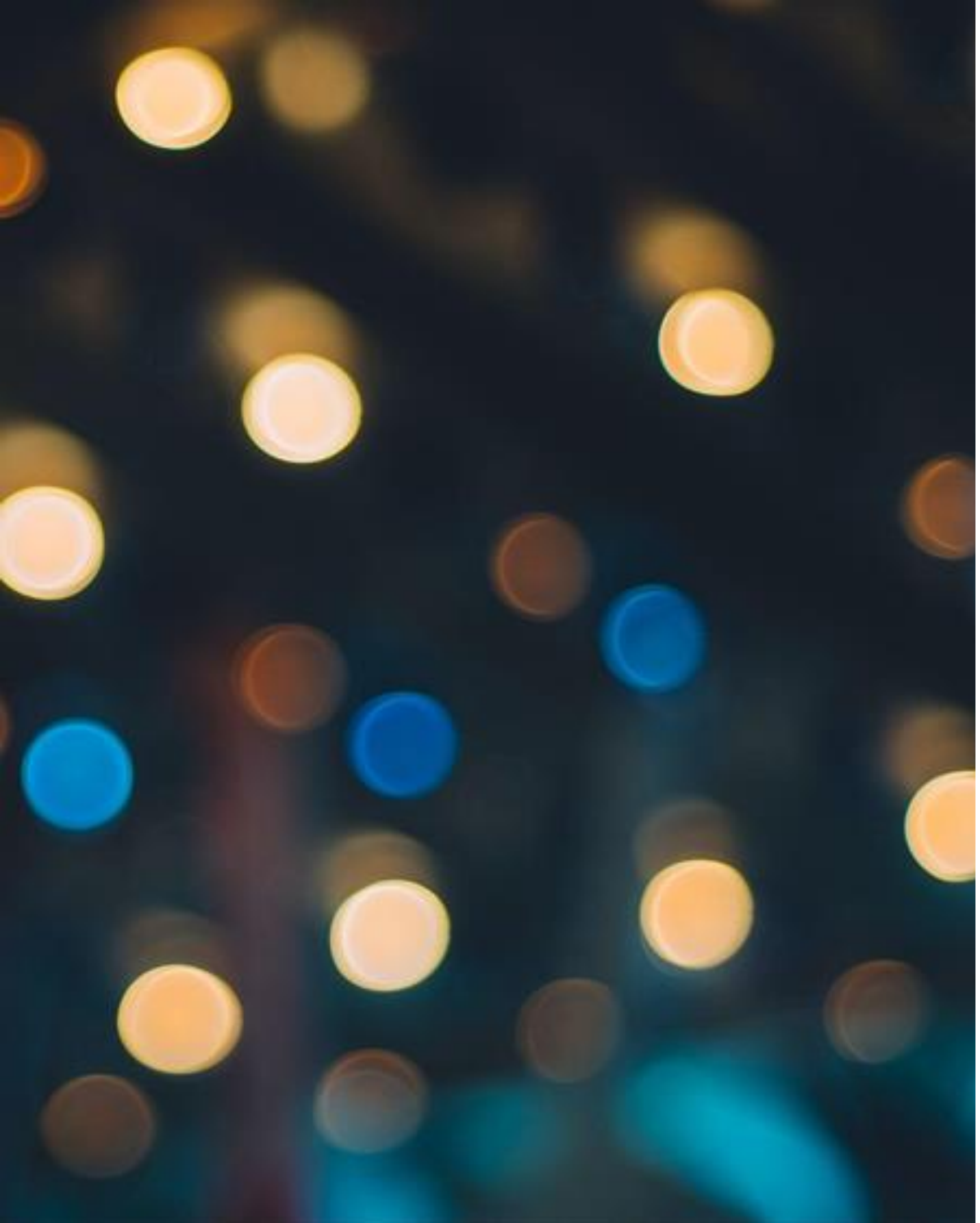

Explanation of tailoring, how to use it, and examples

Tailoring - 3 Steps:

1. **Assess:**
   - Provide context then ask *direct, open-ended questions* to get to know the patient

1. **Acknowledge:**
   - *Verbally acknowledge* or *clarify* the patient's responses

1. **Apply:**
   - Integrate their answers *throughout* the session

GC-PRO

Tailoring Step 1: *Assess*

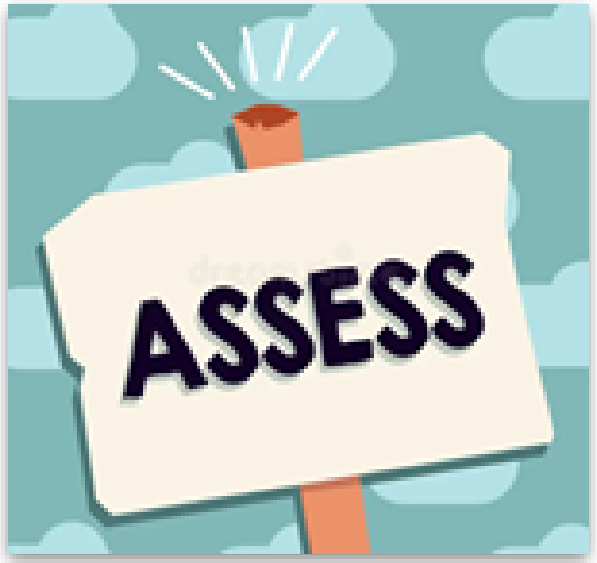

Provide context and ask **key questions** to **get to know the patient**

- **WHEN?** Beginning of the session during typical rapport-building conversation
- *First*, provide some context about what will happen in the session (e.g. topics that can be discussed)
    - This helps patients decide *which* topics are most important to them and what concerns they might have
- ***Then*, ask key questions** to assess the patient's needs

GC-PRO

Tailoring Step 1: *Assess*

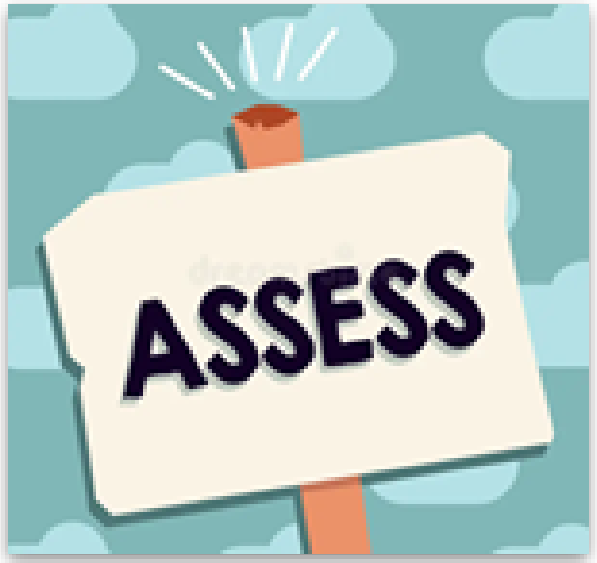

Ask **open-ended, direct questions:**

- “What are your thoughts about genetic testing?”
- “What concerns do you have about ________?”
- “What else might be helpful for you today?
- “What else is on your mind that could be important for today’s appointment?”
- “Which of your beliefs or values related to _____ might be important for me to know?”

Blank examples: “your diagnosis,” “your testing options,” “healthcare”

GC-PRO

Tailoring Step 2: *Acknowledge*

ACKNOWLEDGE

Acknowledge or clarify the patient's response

- **WHEN?** Right after the patient responds to your questions
- Two purposes:
  - Lets the patient know that you **heard** and **accepted** their response - *important for relationship-building*
  - Helps formulate a **mutual agenda** that will successfully meet the patient's needs - *guide and personalize the session*

GC-PRO

Tailoring Step 2: *Acknowledge/Clarify*

Example 1:

Patient: "I do not want to do anything that might put this pregnancy at risk. I've lost two pregnancies before."

Acknowledgement: "Thank you for sharing; that's helpful to know since it will change which testing options we focus on today."

Clarification: "Given your understandable anxiety about pregnancy loss, what might be most helpful to talk about today?"

Tailoring Step 3: *Apply*

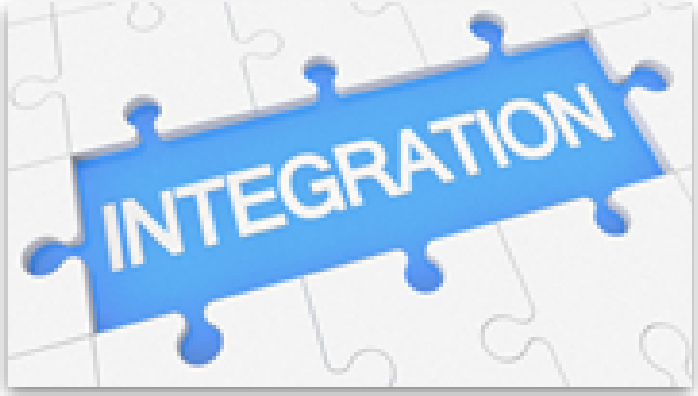

Integrate their answers **throughout** the session

- **WHEN?** Personalize various components throughout the entire session based on expressed needs/values

- Goes hand-in-hand with assess (step 1):
  - *If don't assess needs first, can't tailor the session*
  - *If preferences are not integrated, failure to tailor*

GC-PRO

Tailoring Step 3: *Apply*

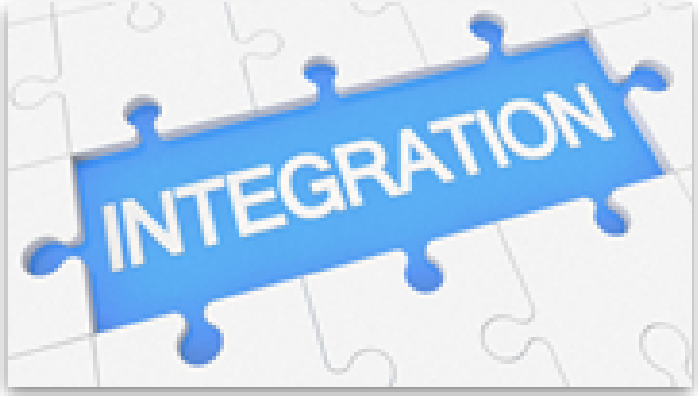

Continued example:

Application 1: “Now, let’s go over your testing options. There are some tests we can talk about that *don’t* put the pregnancy at risk…”

Application 2 (after discussing screening tests): “Other tests we usually offer would give you a more definitive (yes/no) answer, but these other tests come with a risk to the pregnancy. *Based on what you said earlier*, any risk might be too high for you - so what level of detail would you like me to go into regarding the other tests?”

GC-PRO

Tailoring: Putting the steps together

Example 2:

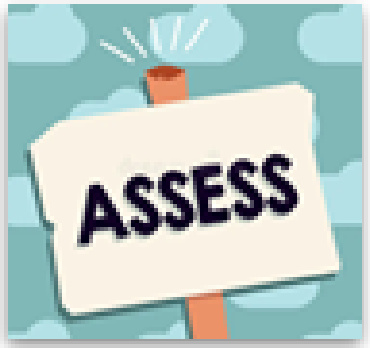

1) Provide context then ask an open-ended, direct question to get to know the patient

Genetic counselor: "So, we've talked about some of the things we'll go over during today's appointment. *What else* is on your mind today that could be important for me to know?"

GC-PRO

Tailoring: Putting the steps together

2) Acknowledge/clarify their response

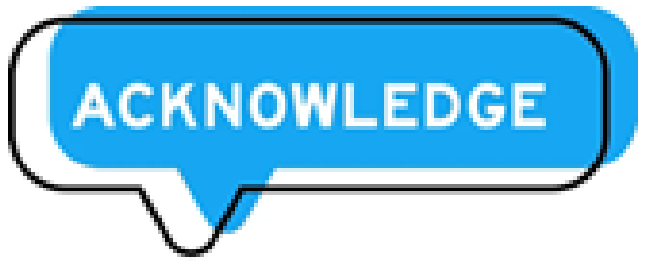

Patient: “I’m very nervous about what this could mean for my grandkids.”

Acknowledgement: ”It’s great you’re thinking ahead about your grandkids’ health.”

Clarification: “What about your grandkids concerns you most?”

Tailoring: Putting the steps together

3) Integrate their response throughout

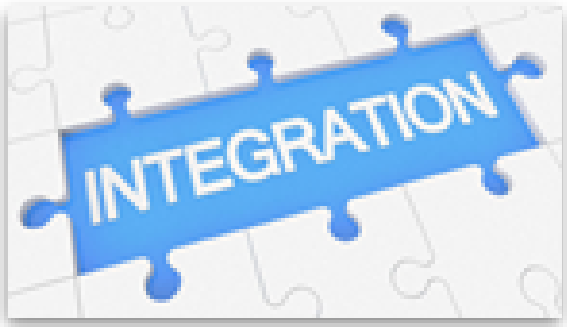

Application 1: "How does this information affect your worries about your grandchildren?"

Application 2: "Grandkids are a big part of your life. How does that impact your decision to do testing?"

GC-PRO

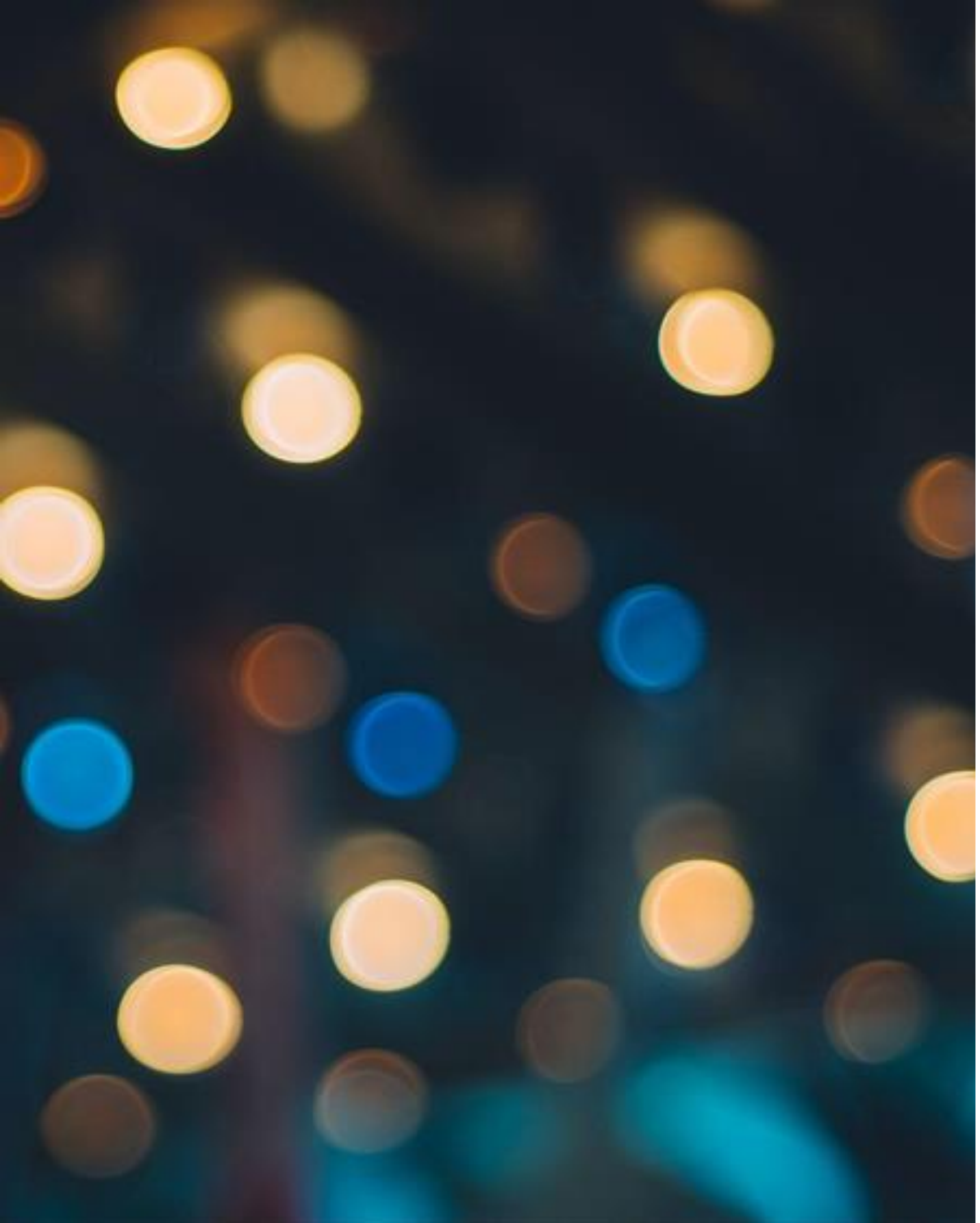

What works for YOU? Decide how to tailor your sessions

GC-PRO

Taylor-swifting

Making space in the session for what each individual really wants and needs.

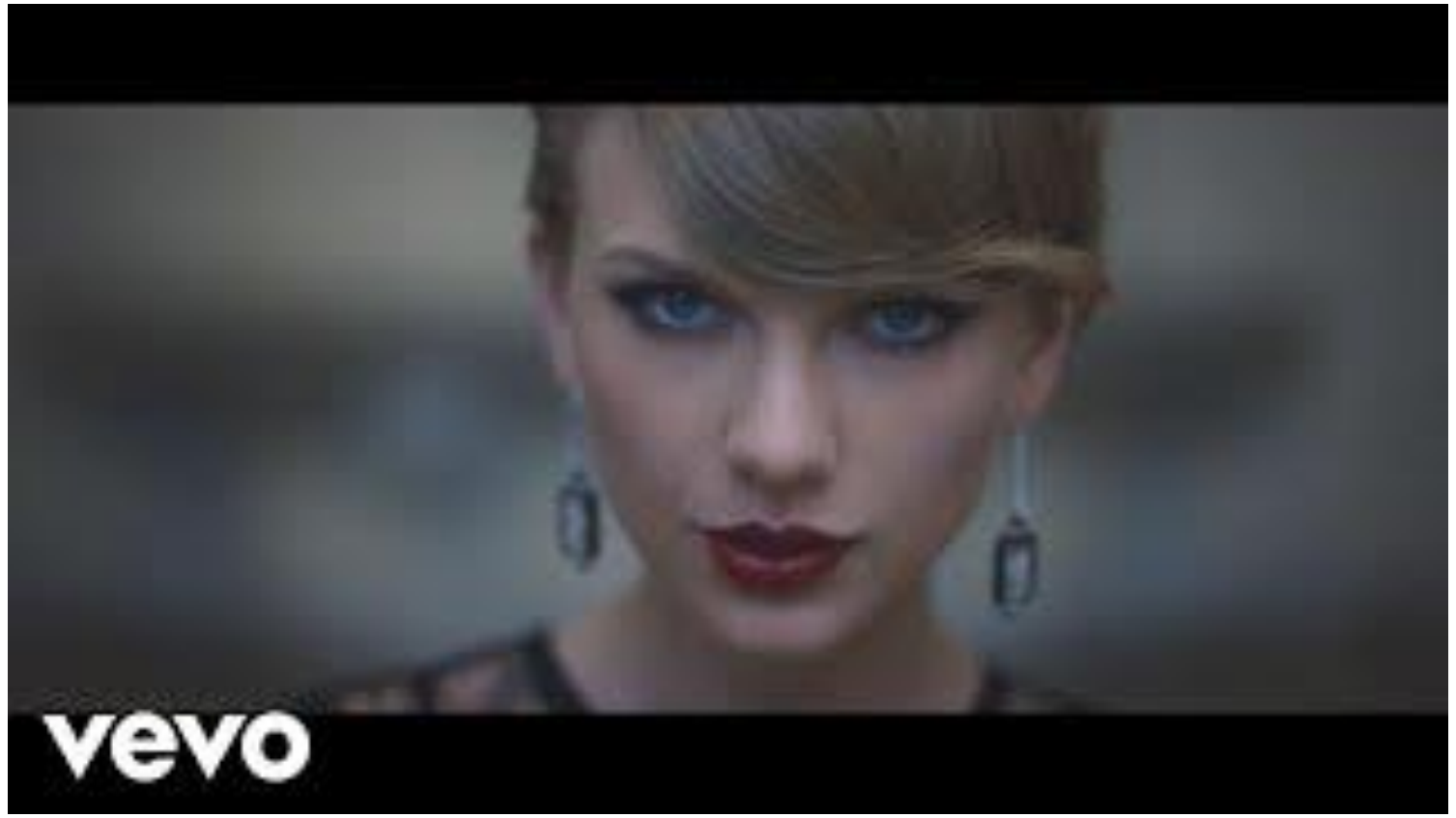

"There's a blank space - and I'll write your name"

Skills Check Time!

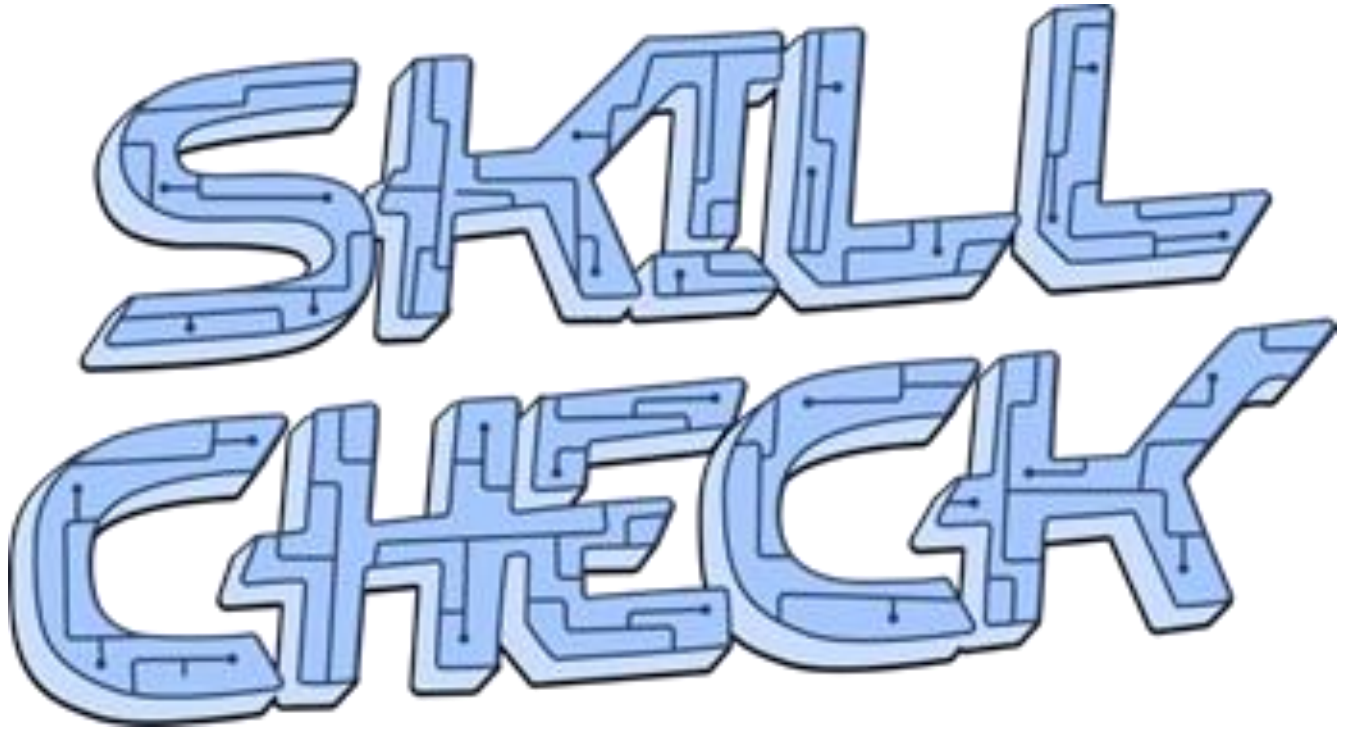

Click on the **"Tailoring - Skills Check"** personalized link from the GC-PRO Training Introduction email.

An interactive form will open on your browser.

Please follow the instructions in the form while viewing these slides.

Skills Check 1: Choose 2 questions

Direct:

- ❑ What concerns do you have today?
- ❑ Which of your beliefs or values could be important for me to know for our visit?
- ❑ What is most important for us to go over today?

What else:

- ❑ *What else* is on your mind today?
- ❑ *What else* might be helpful for you?
- ❑ *What else* would you like to talk about?

OPTIONAL: Come up with your own tailoring questions instead by selecting “other.” Make sure to use a direct or a “what else” question!

GC-PRO

Skills Check 2: There's a blank space... how will you write their name (and elicit their story)?

Think about the 2 questions you chose and **answer the following prompts** within the online form. Your answers should be ~150-200 words.

1. **When will you incorporate these questions** into the flow of your session? And **why** at that point?

1. **How do you think your patients will respond** to these 2 questions? Write out 3 possible reactions.

1. What are some **ways you could incorporate their responses throughout the session?** Write 3 examples showing how you might integrate the patient's response.

GC-PRO

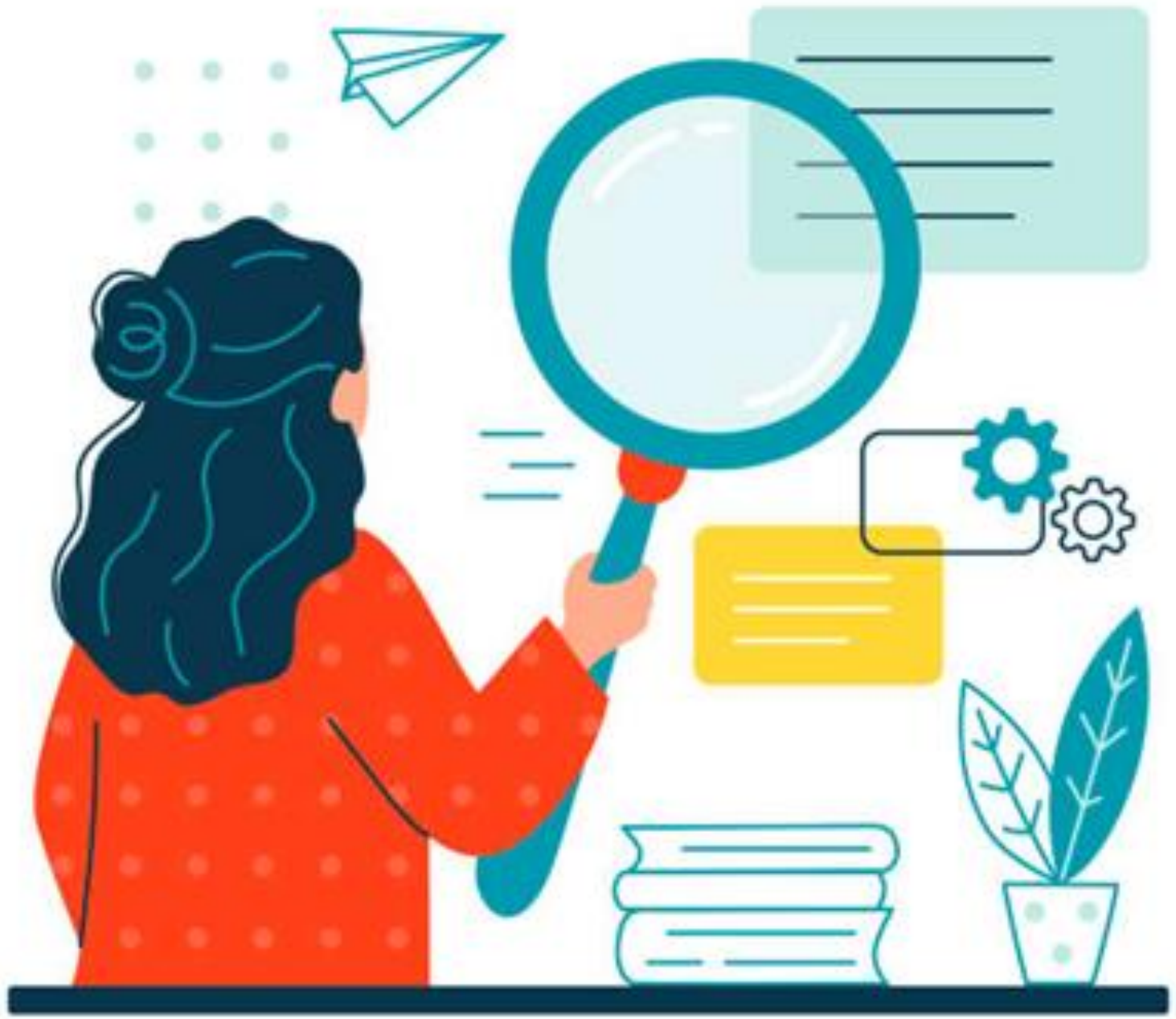

Lastly, how will the researchers be analyzing this skill?

Transparency regarding criteria used to assess this skill

GC-PRO

Criteria for tailoring

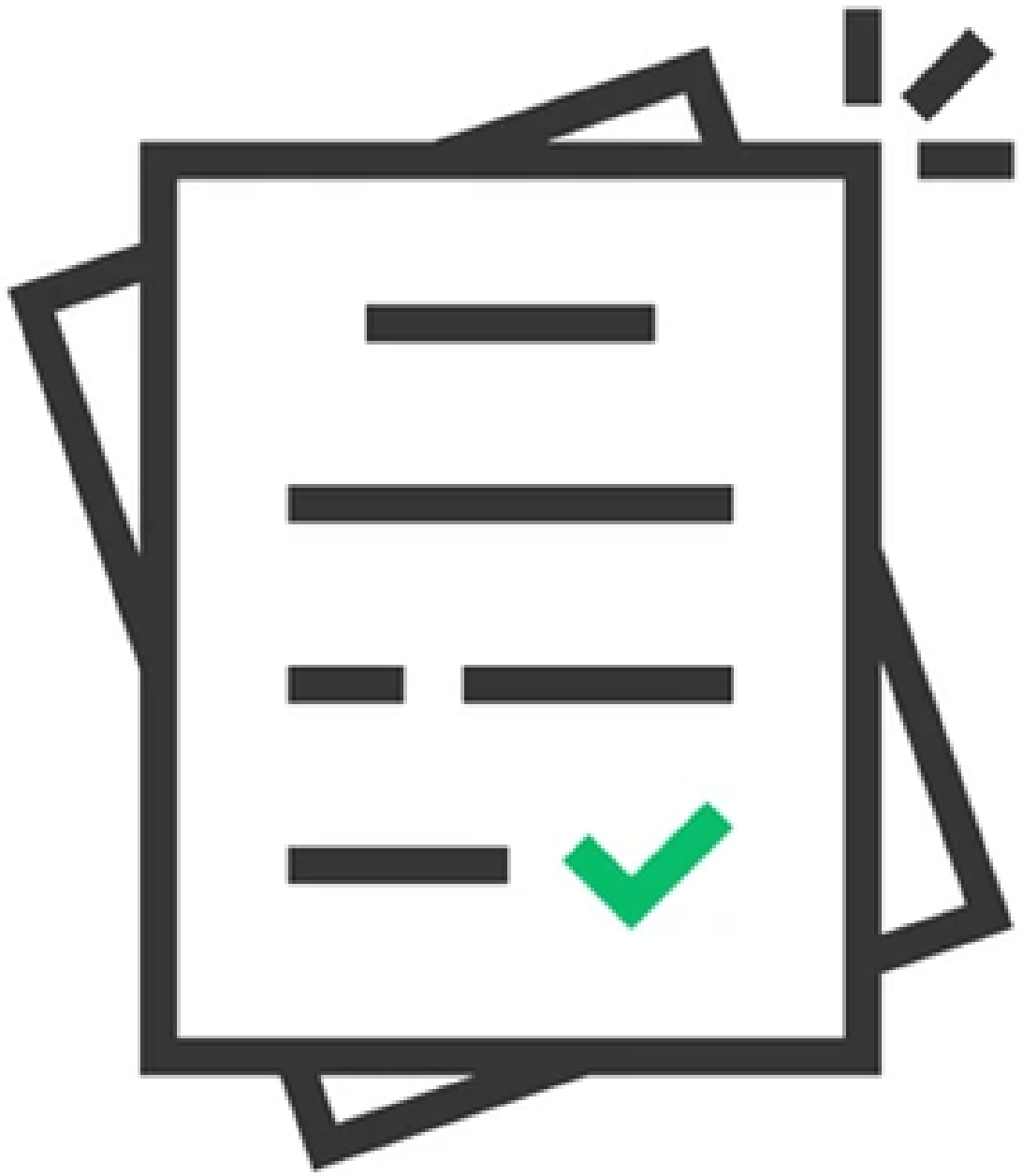

- ❑ Asked **2 or more direct, open-ended** or **“what else” questions** related to the patient’s needs and “getting to know them” at the beginning of the session
- ❑ **Acknowledged/clarified** the patient’s needs and responses
- ❑ **Integrated** the patient’s responses (e.g. concerns, values, what’s important) **at least 2 times** in the session

GC-PRO

Taylor swifting is as easy as:

1) Assess
2) Acknowledge
3) Apply

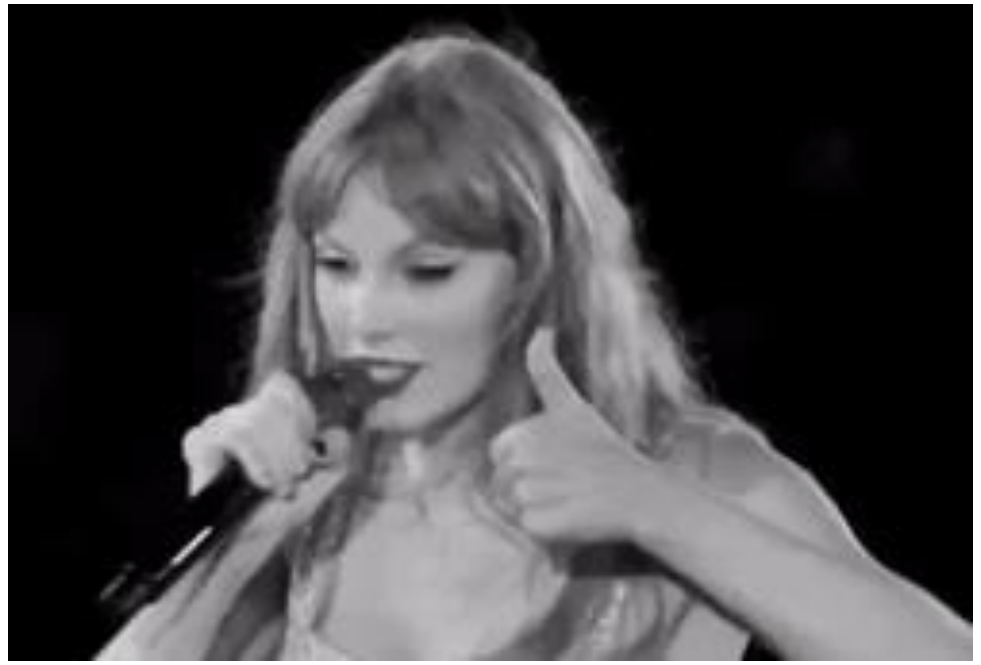

There's always a blank space to incorporate your patients' needs & values!

GC-PRO

Final instructions

1. After completing the 2 skills checks in the online form, please press **submit** to record your answers.
   a. We recommend you **download a PDF of your responses** to save for next week's discussion (click on the button at the bottom of the submission page).

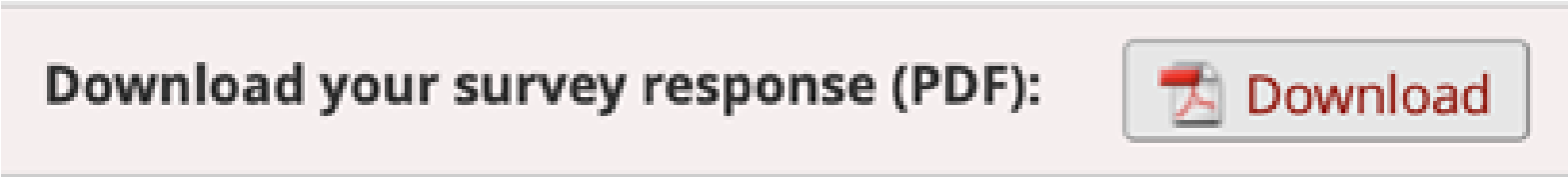

1. **Continue onto the next skill**, "Streamlining Information Gathering", and complete the corresponding skills checks.

GC-PRO

Thank you!

Heather Zierhut:
zier0034@umn.edu

Debi Cragun:
dcragun@usf.edu

Elena Fisher:
fishe912@umn.edu

GC-PRO

References

- Redlinger-Grosse, K., MacFarlane, I. M., Cragun, D., & Zierhut, H. (2021). A Delphi study to prioritize genetic counseling outcomes: What matters most. Journal of Genetic Counseling, 30(3), 676-692.
- *(Standardized patient quotes):* Manuscript in press at Journal of Genetic Counseling: Cragun et al., "Unveiling genetic counseling skills: Developing an online training course and analyzing counselor communication practices" (2025)
- Allgood, S., Park, J., Soleiman, K., Saha, S., Han, D., McArthur, A., ... & Beach, M. C. (2023). Taxonomy and effectiveness of clinician agenda-setting questions in routine ambulatory encounters: A mixed method study. Patient Education and Counseling, 115, 107889.
- Unpublished data from the Genetic Counseling Processes Result in Outcomes (GC-PRO) study, stage 1

GC-PRO

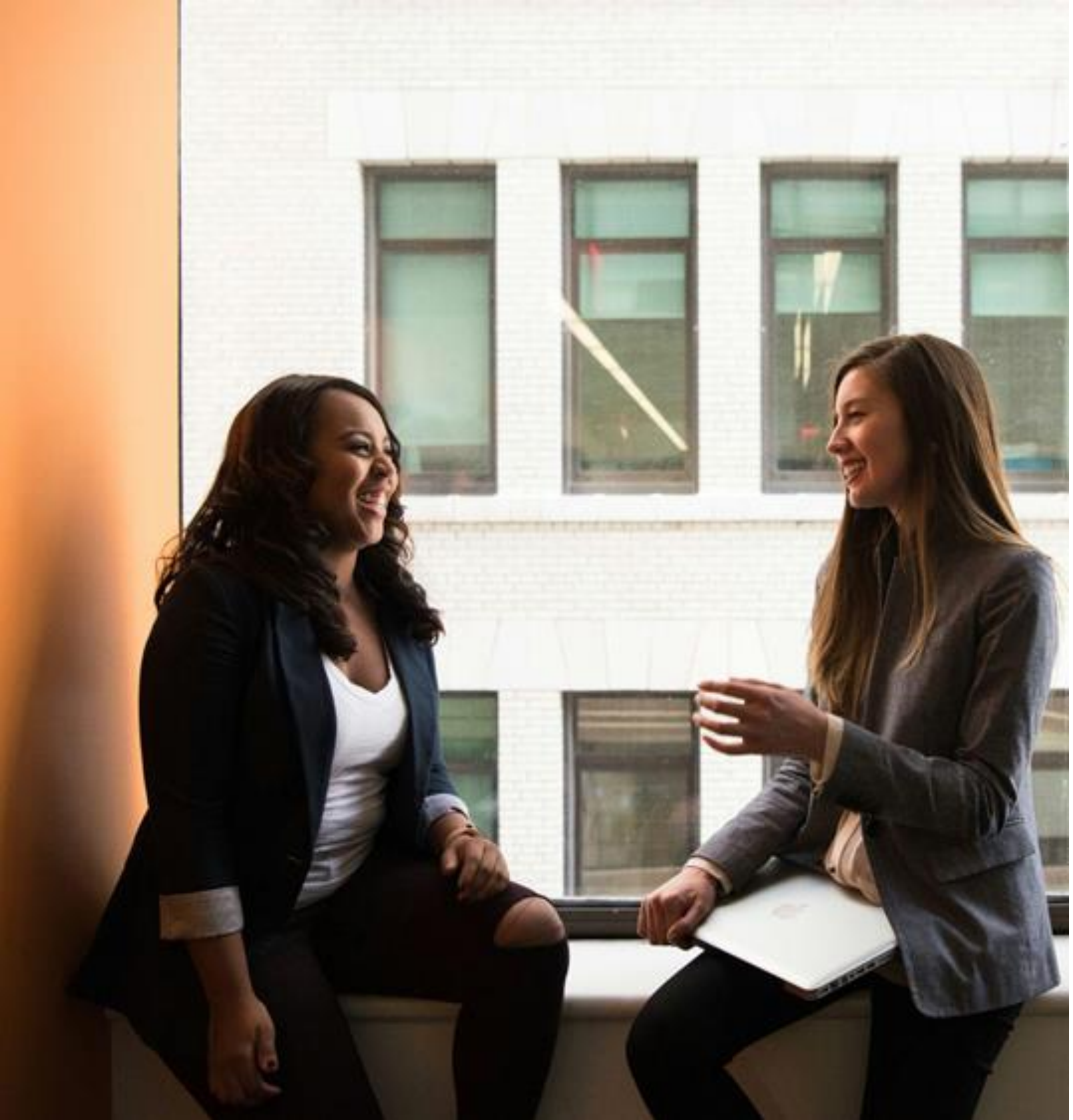

Genetic Counseling Processes Result in Outcomes:

Streamlining Information Gathering

Skill #2

Week 1

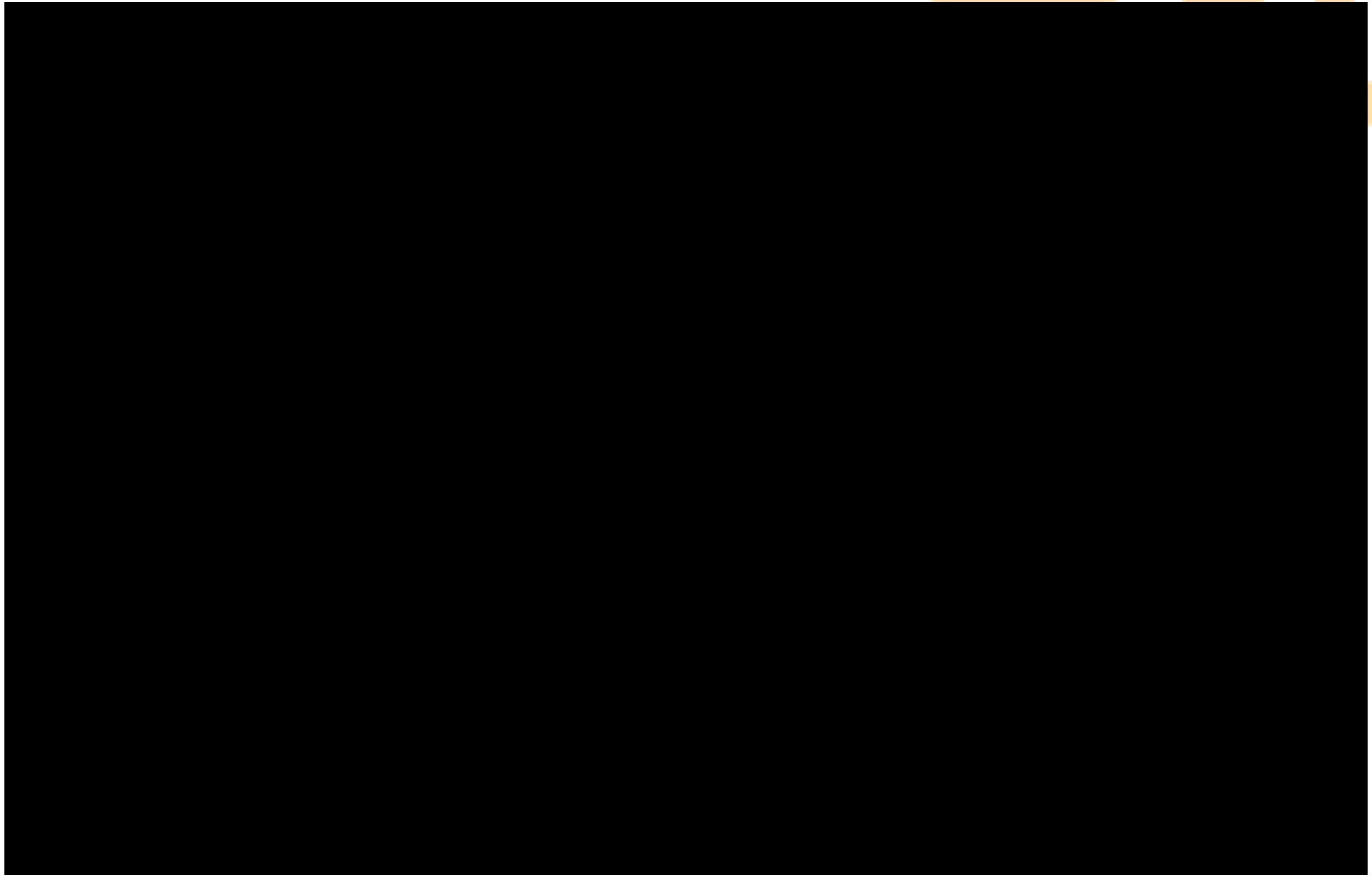

Watch this video to review content together,
or move through the slides at your own pace!

GC-PRO

Confidentiality reminder:

Please do not share the content of any GC-PRO training materials with anyone, including other participating GC-PRO genetic counselors at your institution. Thank you!

GC-PRO

Outline

1. Skill overview & why streamlining matters
2. Ways to streamline
3. Decide how you will "Explain why & Streamline" in your sessions

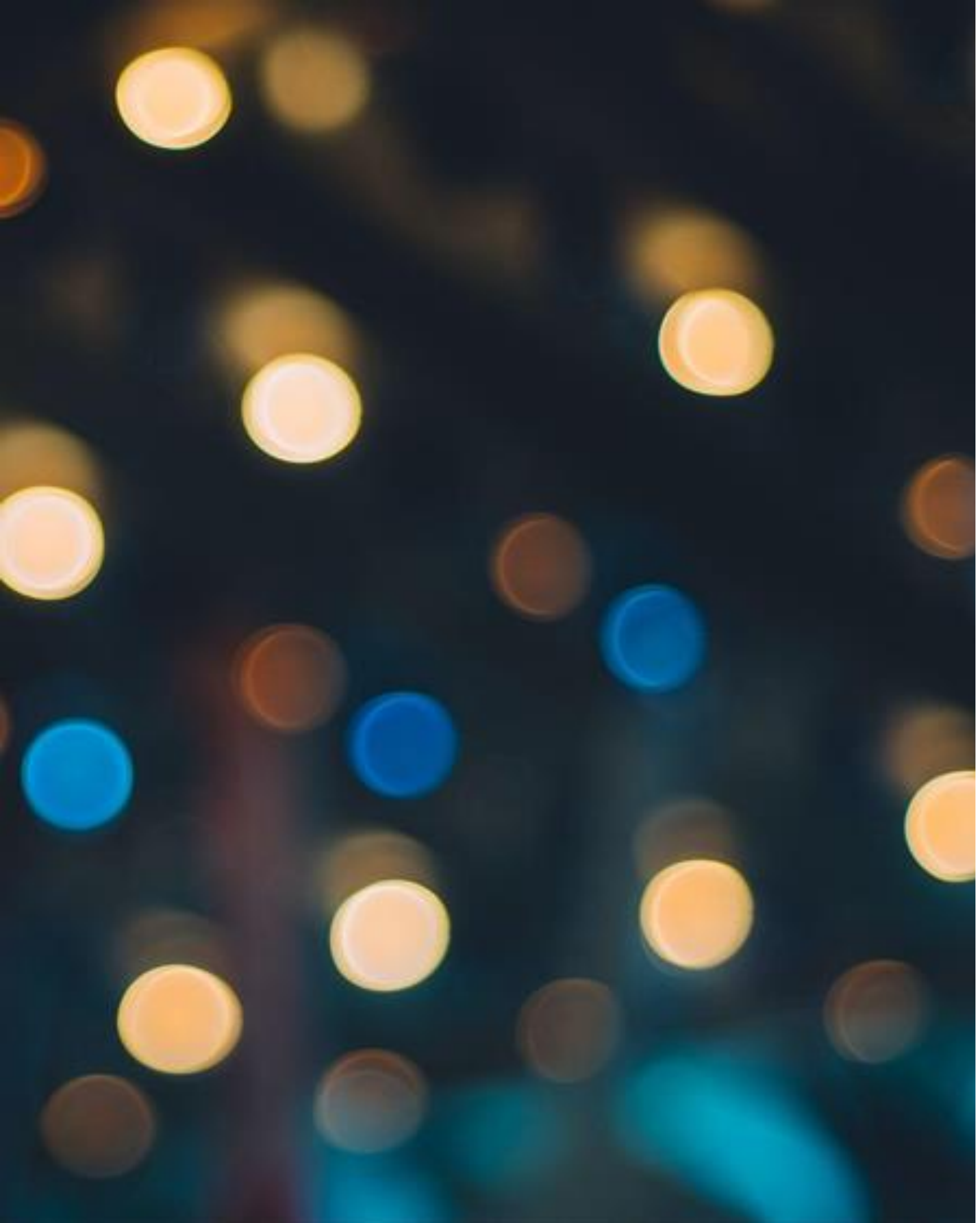

Rationale for use and evidence behind the skill

GC-PRO

Streamlining Information Gathering - Overview

Streamlining means:

- **Limiting questions** to elicit medical and family history information that could substantially impact:
  - Patient decision-making
  - Tests that are offered
  - Case management

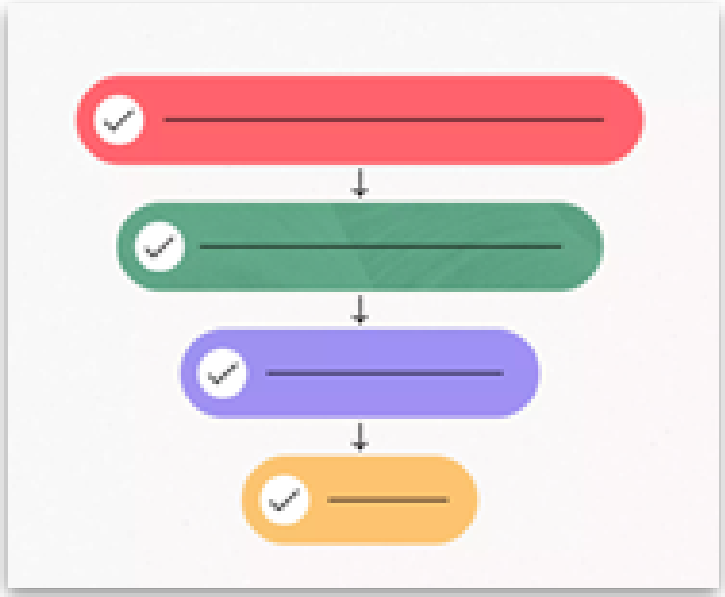

- **Redirecting** conversations to more important or relevant topics

GC-PRO

Why streamline?

Increases **efficiency** & **avoids overwhelming** patients

- The **amount of time spent gathering information** was one of the main factors that increased session time in GC-PRO part 1.
- Some GC-PRO patients thought too many questions were asked and **disliked** that the counselor **never used all the information**.
- Standardized patients rated sessions **higher** if they were ***not bombarded*** with medical and family history questions, especially at the start of the session.

GC-PRO

Why streamline?

Importance of **efficiency:**

- GC-PRO patient **quality** ratings were **NOT** associated with session time
- ***Longer*** sessions had ***lower*** patient empowerment and activation scores
- Patient activation increased ***less often*** among overly talkative patients

GC-PRO

Why streamline?

Helps to **focus** and **tailor** the session

- Patients prefer when **information is integrated** from before, during, and after the session *(manuscript under review at JoGC)*
  - *Example:* Gathering relevant information in a pre-session intake and then APPLYING the info collected to the session
- Standardized patients found it helpful when the information gathered was *directly used* to **tailor and personalize** the session *(manuscript in press at JoGC)*

GC-PRO

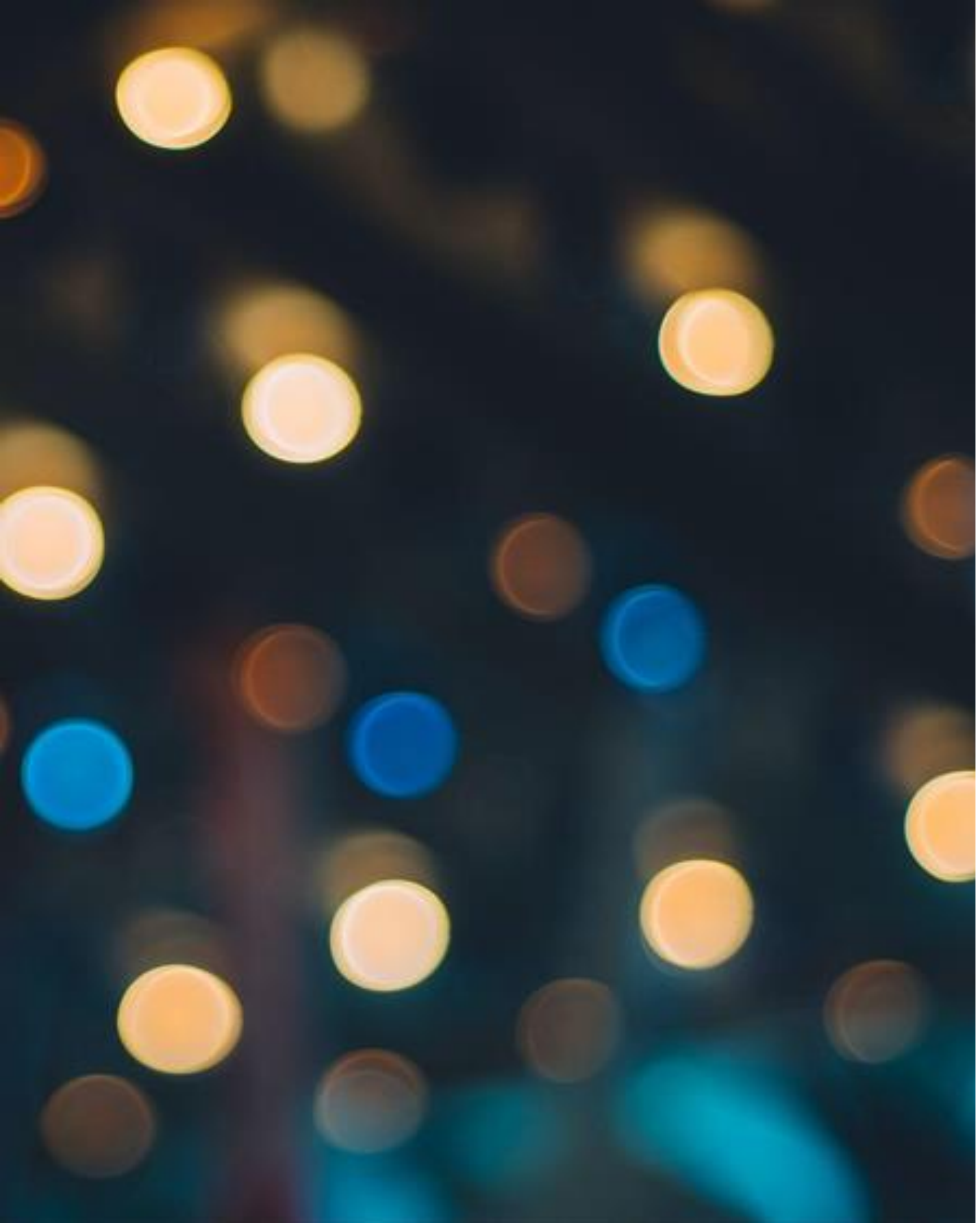

Explanation of streamlining, how to use it, and examples

GC-PRO

Streamlining Info Gathering

- Prioritize asking the *most important information* to **improve efficiency**, **support** tailoring of the session, and **avoid** patient **disengagement**

- **WHEN?**
  - BEFORE the session, prioritize which questions to ask
  - IN the session, limit the number of questions asked during:
    - Medical history and family history intake
    - Risk assessment

GC-PRO

Prioritizing questions

- When deciding whether to adjust your intake workflow, consider the following **"What + How + Why"** criteria:

What do you *absolutely* need to know about the patient to identify their *relevant* medical issues and concerns?

How might knowing this information *change* what you do for the patient?

Why would collecting this information *help* the patient?

GC-PRO

Prioritizing questions

Setting yourself up for success:

(We will practice this in the Skills Checks)

KEEP: Prioritize information needed to take action (i.e., questions that *meet* the "What + How + Why" criteria)

MAYBE: Consider asking questions that *don't* meet these criteria, or may only be needed for certain scenarios or relatives

GET RID OF: Eliminate all other questions

GC-PRO

Streamlining - 2 Steps:

1. **Explain why:**
   - Explain *how or why* the information you're collecting is being used in the session

1. **Streamline:**
   - 2a) Ask only pertinent questions
   - 2b) *IF NEEDED:* Redirect patients with "Yes, AND"

GC-PRO

Streamlining Example: Prenatal setting

Step 1 - Explain why: "I am going to collect some information about your personal and family history to determine risks for your current pregnancy."

Step 2a - Which questions are MOST important for this indication?

- Family members with cancer & types ?
- Medication use in pregnancy ✓
- Birth defects ?
- Skin conditions ✗
- High cholesterol or blood pressure ?
- History of miscarriages ✓
- Learning difficulties / developmental delays ?
- Dementia ✗

- Drawing out 1st-degree relatives ✓
- Drawing out all 2nd-degree relatives ?
- Drawing out 3rd-degree relatives ?
- Ages of each family member ?

GC-PRO

Streamlining Example: Cancer setting

Step 1 - Explain why: "I'm going to ask about your family because different types of cancers and ages of diagnosis can give clues about whether there may be a cancer causing gene in your family."

Step 2a - Which questions are MOST important for this indication?

- Family members with cancer, types of cancer ✓
- Ages at cancer diagnosis ✓
- Alcohol use ?
- Birth defects ✗
- Skin conditions ?
- Age at menarche and menopause ?
- Breast density ?
- History of miscarriages ✗
- Learning difficulties / developmental delays ?
- Ages of each family member ?
- Cancer screenings (skin, breast, colon) ?
- Prior hx of breast biopsies ✓
- Drawing out 1st-degree relatives ✓
- Drawing out all 2nd-degree relatives ?
- Drawing unaffected 3rd-degree relatives ✗

GC-PRO

Streamlining Example: General genetics

Step 1 - Explain why: "I'm going to ask some questions about your personal and family history to help us understand whether the cause of your [symptoms/reason for referral] could be genetic."

Step 2a - Which questions are MOST important for this indication?

- Family members with cancer & types ?
- Pregnancy history (complications/exposures) ?
- Birth defects ?
- Skin conditions ?
- High cholesterol ?
- High blood pressure ?
- History of miscarriages ?
- Developmental history ?
- Learning problems ?

- Drawing out 1st-degree relatives ✓
- Drawing out all 2nd-degree relatives ?
- Drawing 3rd-degree relatives ?
- Ages of each family member ?

GC-PRO

Streamlining - 2 Steps:

1. **Explain why:**
   - Explain *why* or *how the* information you're collecting is being used in the session

1. **Streamline:**
   - 2a) Ask only pertinent questions
   - 2b) *IF NEEDED:* Redirect patients with "Yes, AND"

GC-PRO

Redirecting Patients When Needed...

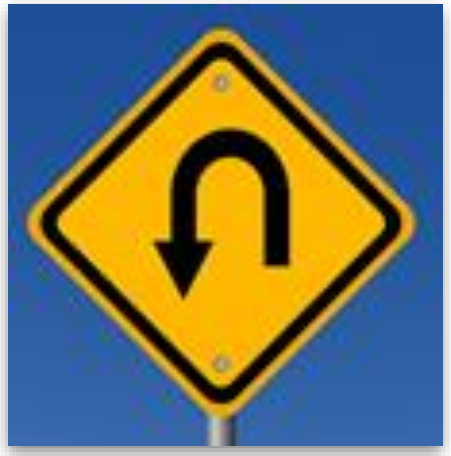

Use **"Yes, AND..."**

- Acknowledge patient's statement first to ensure they feel heard, then say **AND immediately**
- After saying **AND** you can:
  - Ask a new question
  - Indicate where you want to go next
  - Explain why you are moving on
  - Suggest a way to get their other needs met, while focusing them on *your* role and expertise

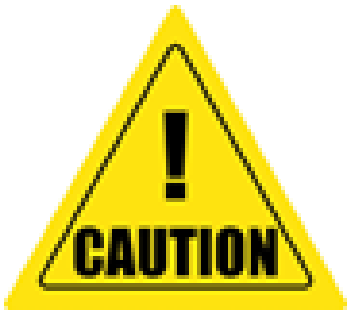

CAUTION: *"Yes, BUT"* negates what they said

GC-PRO

Streamlining using “Yes, AND” examples

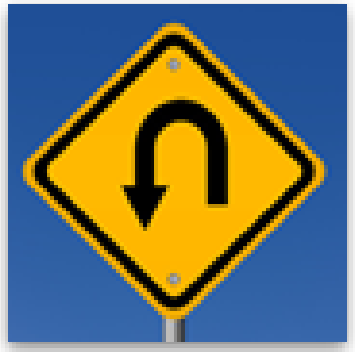

“Thank you for that information **AND** I’m wondering if you could tell me more about____?”

“That seems challenging **AND** it may be helpful if we_____”

“I can see you care about _____ **AND** I want to be sure we also have time to discuss the concern you had about ____.”

“That is a great topic to talk more about with your oncologist (or Ob/Gyn) **AND** today perhaps we can focus more on the genetics part, since that is where I can be most helpful.”

GC-PRO

Other Ways to Streamline

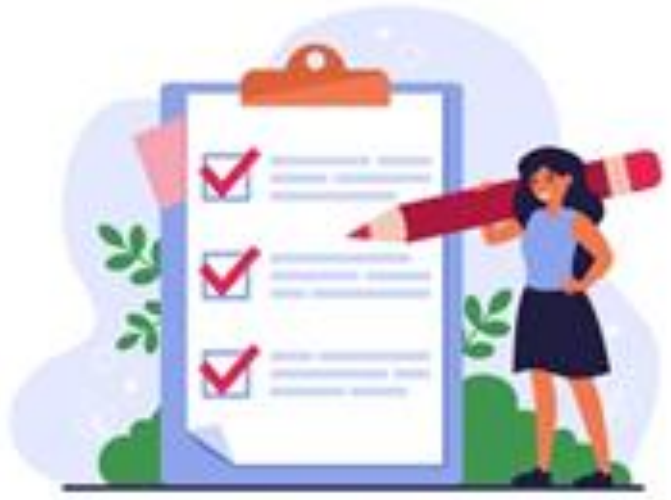

1. Move questions to **pre-visit questionnaires**
2. Ask **closed-ended** questions when gathering information
3. **Explain *exactly*** what you are looking for in the family history
4. Do NOT ask about the health of every family member separately
5. Ask which conditions/risks they are most concerned about to help **prioritize and streamline** your discussion

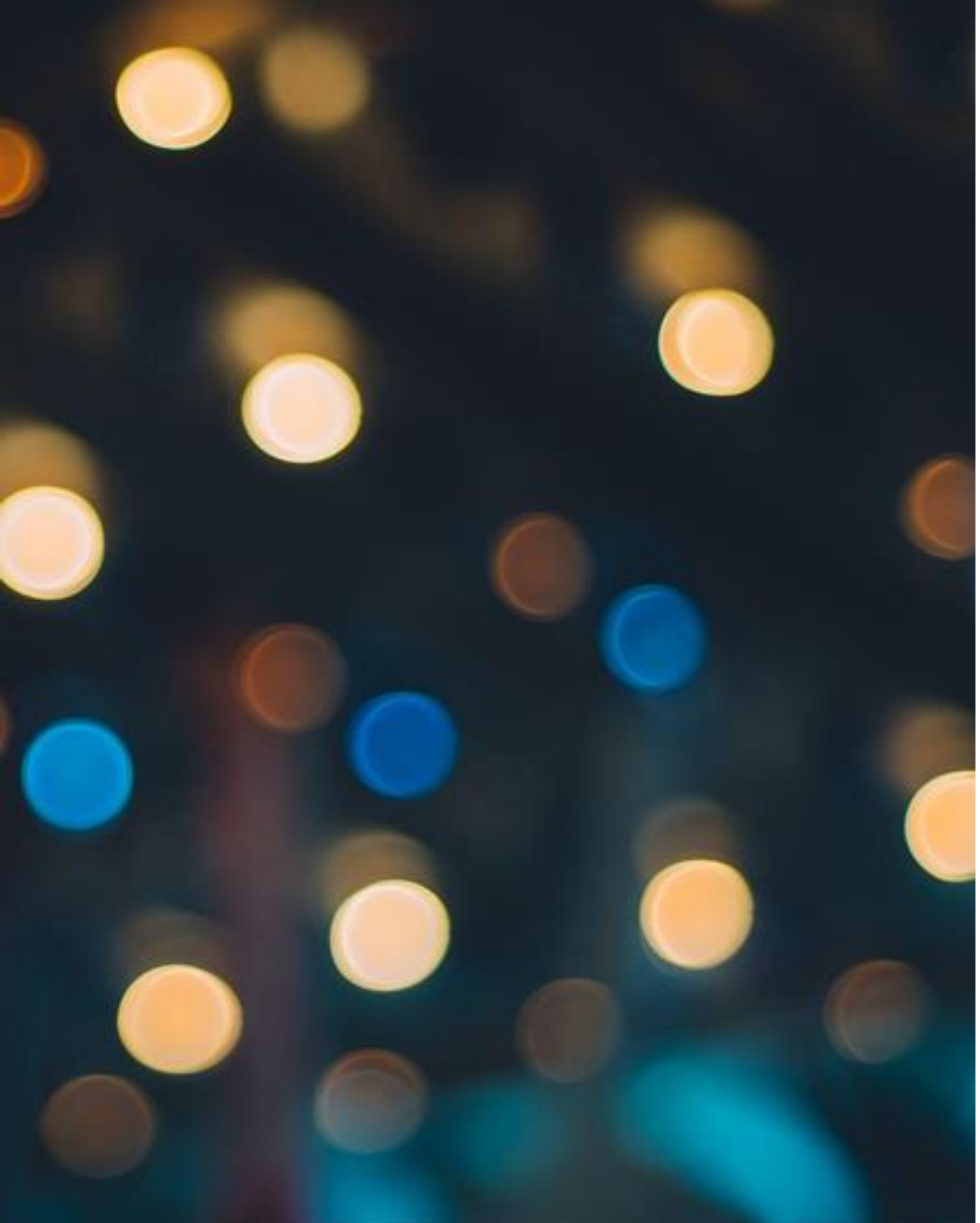

What will work for YOU? Decide how to streamline your info gathering

GC-PRO

Streamlining

"Explaining WHY," maybe, BYE BYE BYE

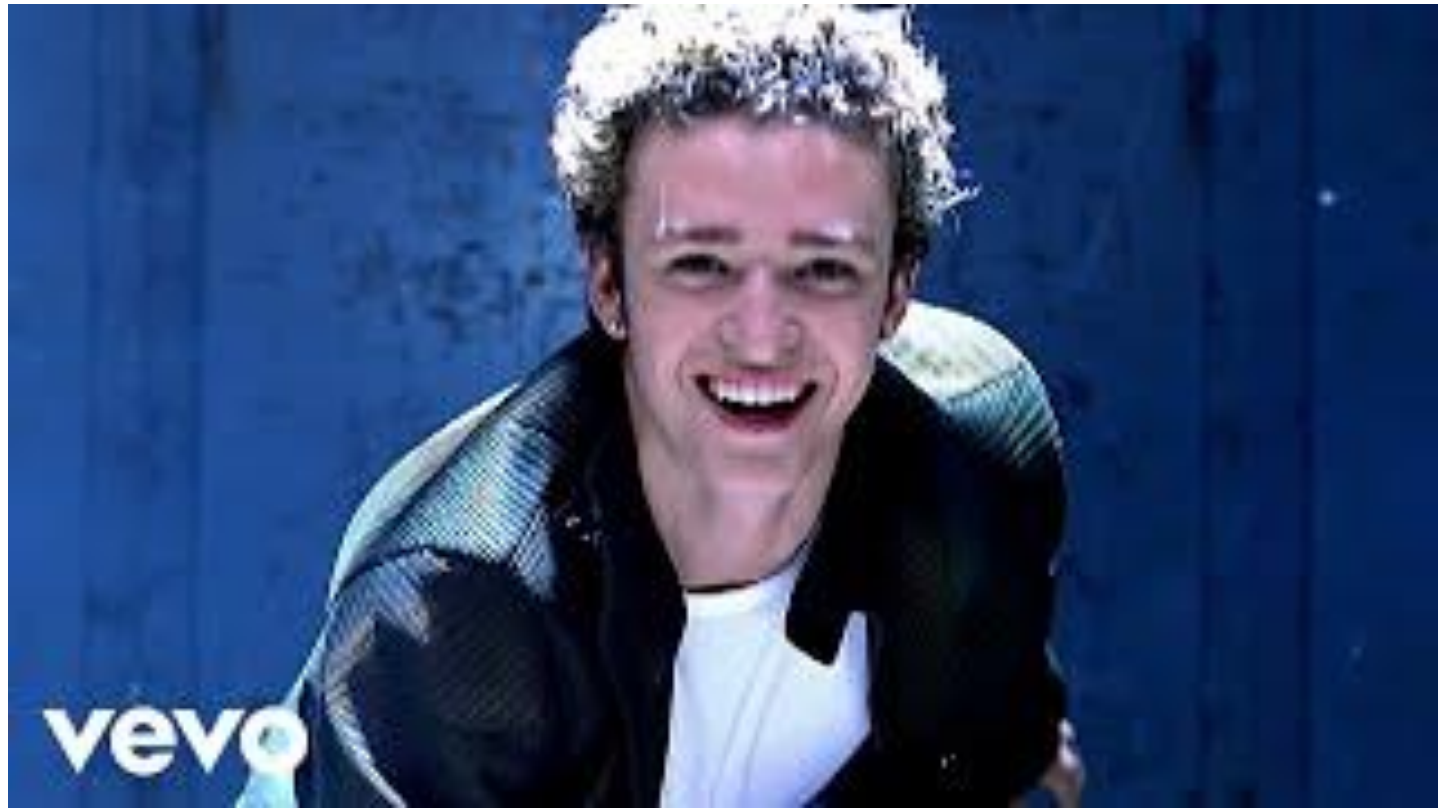

It ain't no lie, patients want you to say *bye bye bye* ***to all those questions!***

GC-PRO

Skills Check Time!

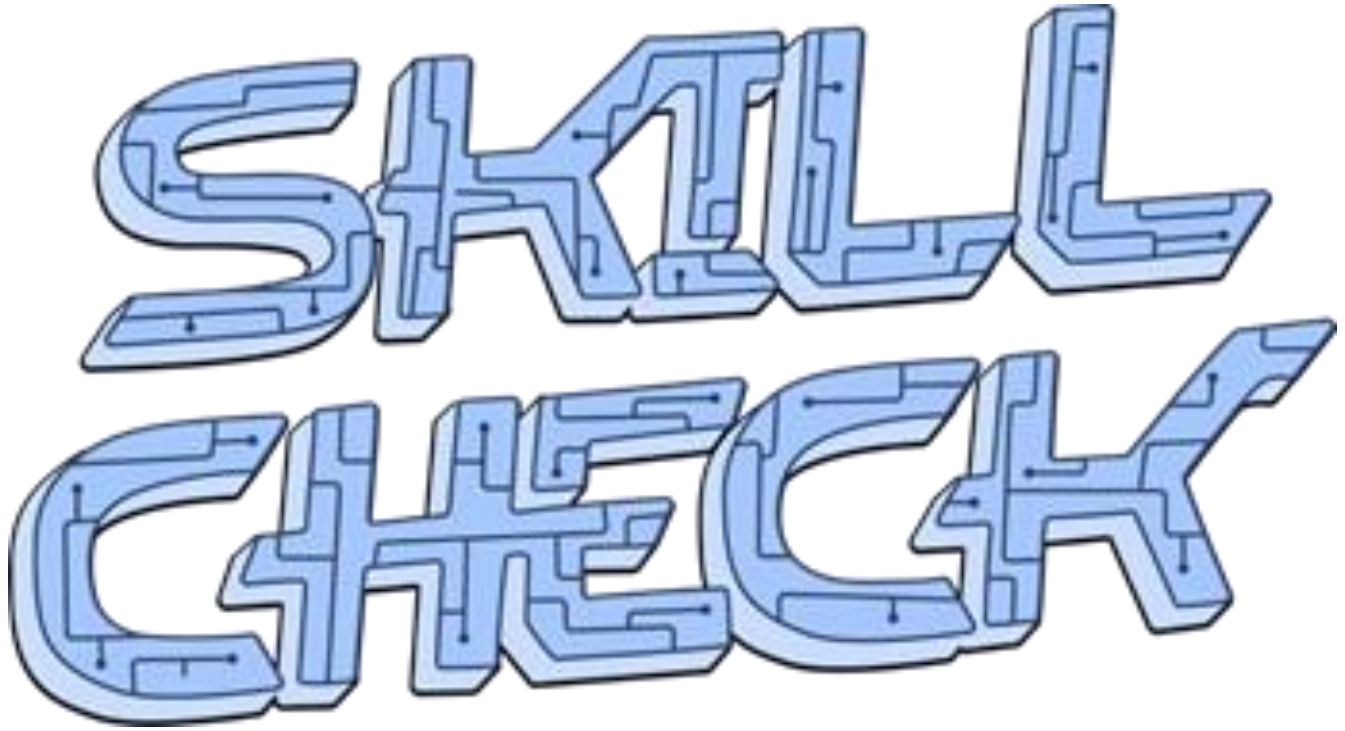

Click on the **“Streamlining - Skills Check”** personalized link from the GC-PRO Training Introduction email.

An interactive form will open on your browser.

Please follow the instructions in the form while viewing these slides.

GC-PRO

Skills Check 1a: Taking inventory

1. **Start by writing out out** all of the intake questions you routinely ask for your *most common* indication(s), including:
   a. Personal medical history questions
   b. Family history collection
   c. Other risk assessment questions

1. Count the **total number** of routine questions asked for 1 indication.

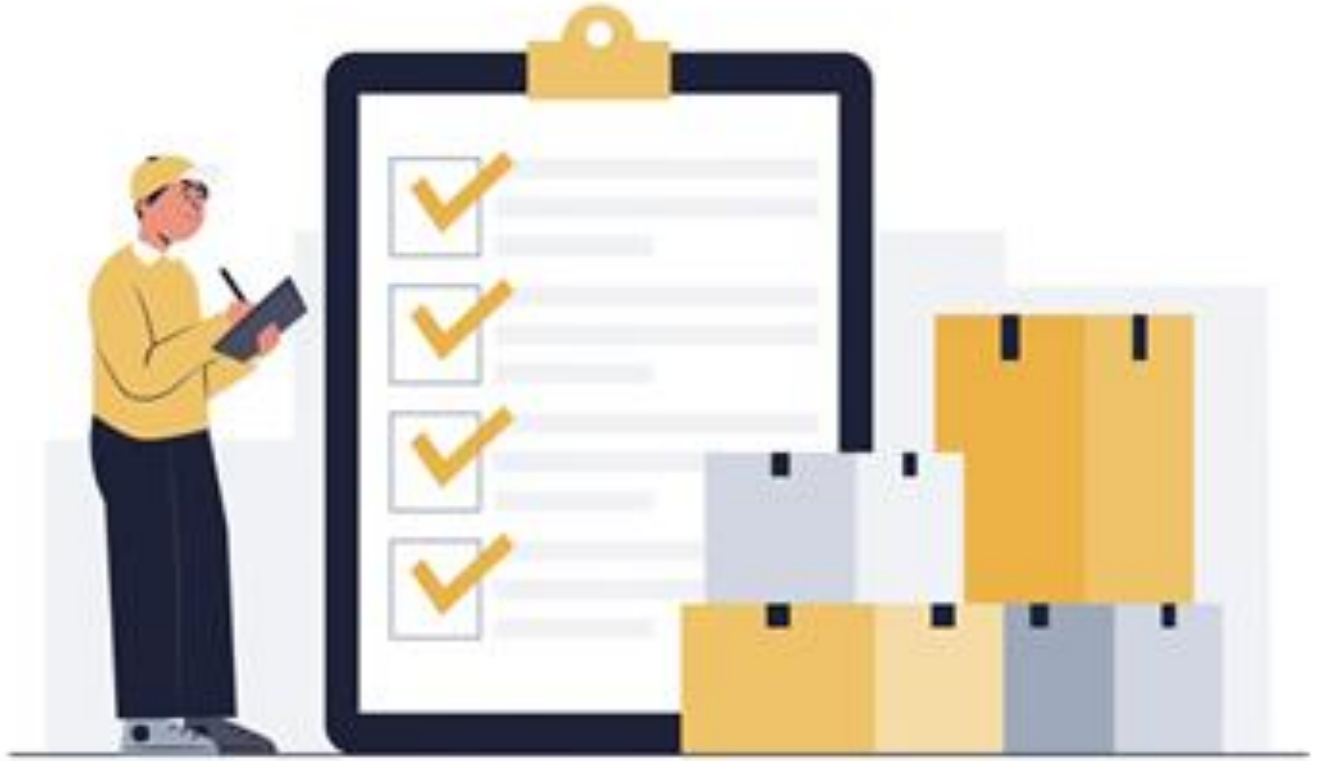

Skills Check 1b: Sorting and Prioritizing

3. Sort your intake questions based on the **"What + How + Why"** criteria:

What do you *absolutely* need to know to identify the *primary* medical issue/concern?

How (and how much) will knowing this information *change* what you do for the patient?

Why would collecting this information *help* the patient? (Patients like to know why too)

✓ **KEEP:** High priority questions that meet all 3 criteria above

? **MAYBE:** Meet some criteria or might be needed in certain situations

✗ **Bye, Bye, Bye:** Do not meet any criteria or are less important

Skills Check 1c: Refining

Reflect on what else may impact your question prioritization:

4. Pick **2** questions from the **KEEP** pile (if it is still long) or from the **Maybe** pile.
5. Answer the **prompts** below to determine if their categorization changes:
   a) For which cases will this question be *critical* and which cases is it *not*?
   b) How much does this question *change* the risk assessment or differential diagnoses?
   c) Does the question *need* to be asked to avoid medical liability? Why or why not?
   d) What is an alternative strategy (examples on slide 20) could you use to gather this information instead? Select 2 or more and discuss feasibility of using these strategies.

Skills Check 2: Redirecting

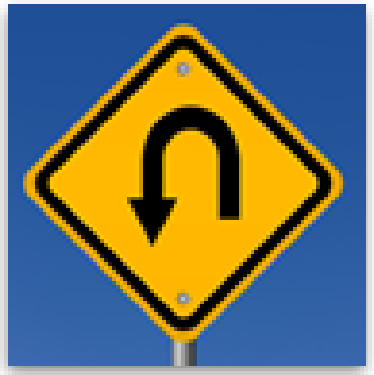

Please answer the following prompts in the online form:

1. Identify a time when you could have better redirected a talkative patient or when you may have simply interrupted a talkative patient. **Describe the scenario** - including what you did and how the patient reacted.

2. **Would the use of "Yes, AND" have helped** in that situation? Why or why not?

3. What **other ways** have you found to **redirect** patients, but still ensure they feel like they can **share their story** and what is important to them?

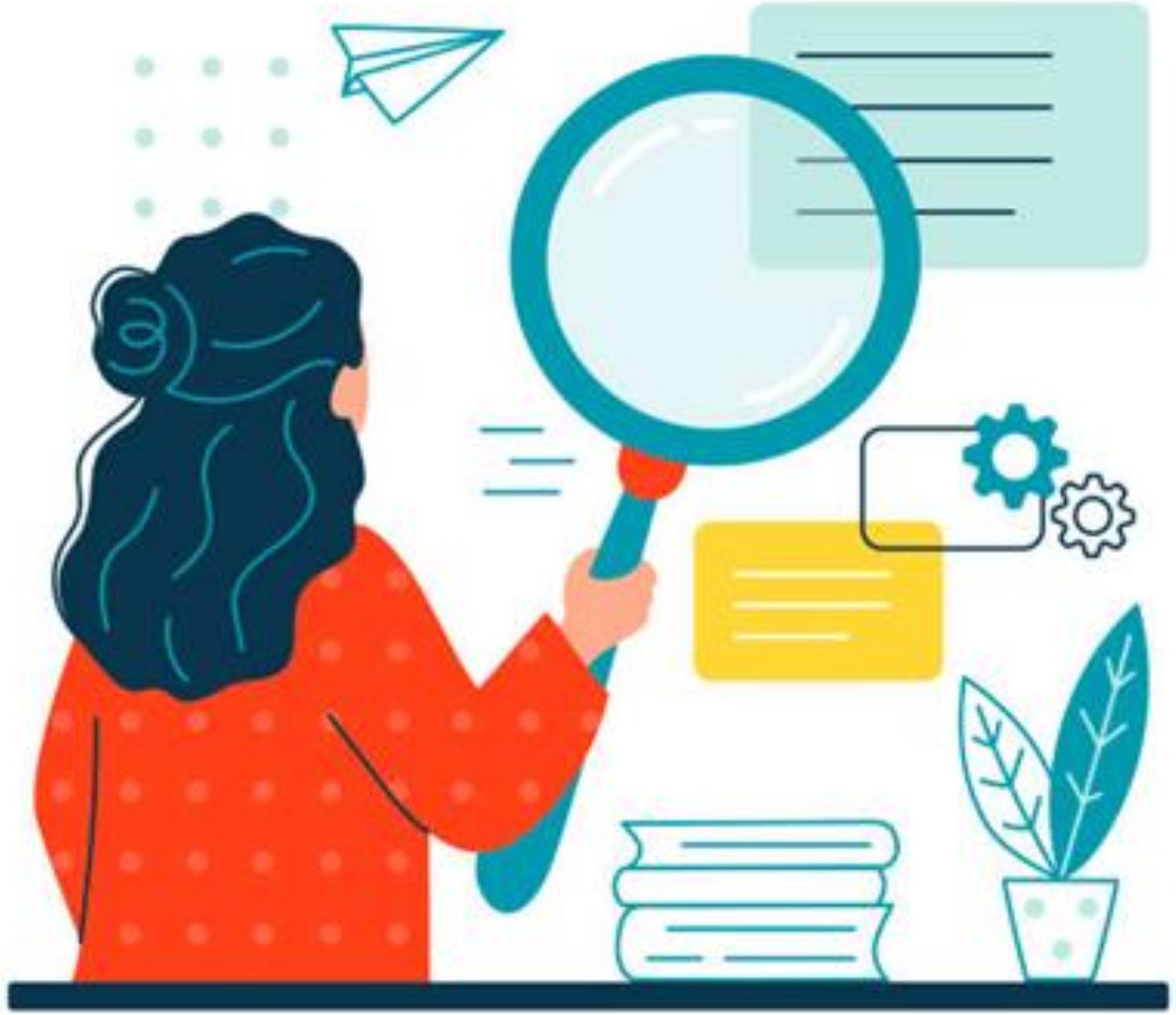

Lastly, how will researchers analyze this skill?

Transparency regarding criteria used to assess skill

Criteria for streamlining info gathering

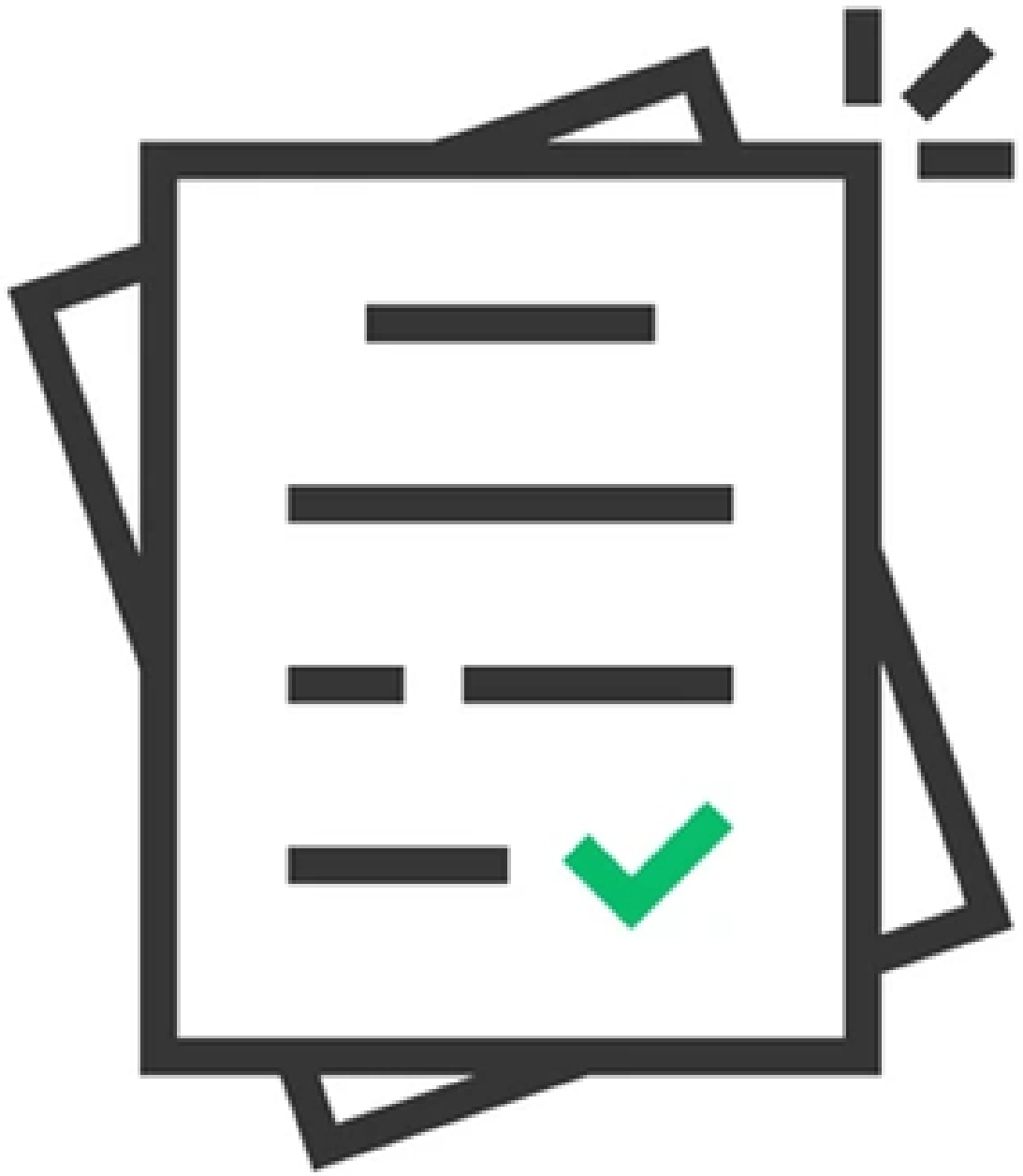

- ❑ **Explain Why:** Completed or not completed
- ❑ **Compare percentage of time in information-gathering** with other parts of the session (before vs. after training)
- ❑ **Use of “Yes, AND”:** Completed or not completed when needed

Streamlining is as easy as:

Explaining WHY

Saying **BYE BYE BYE** to extra questions

Using **"Yes, AND"** when needed

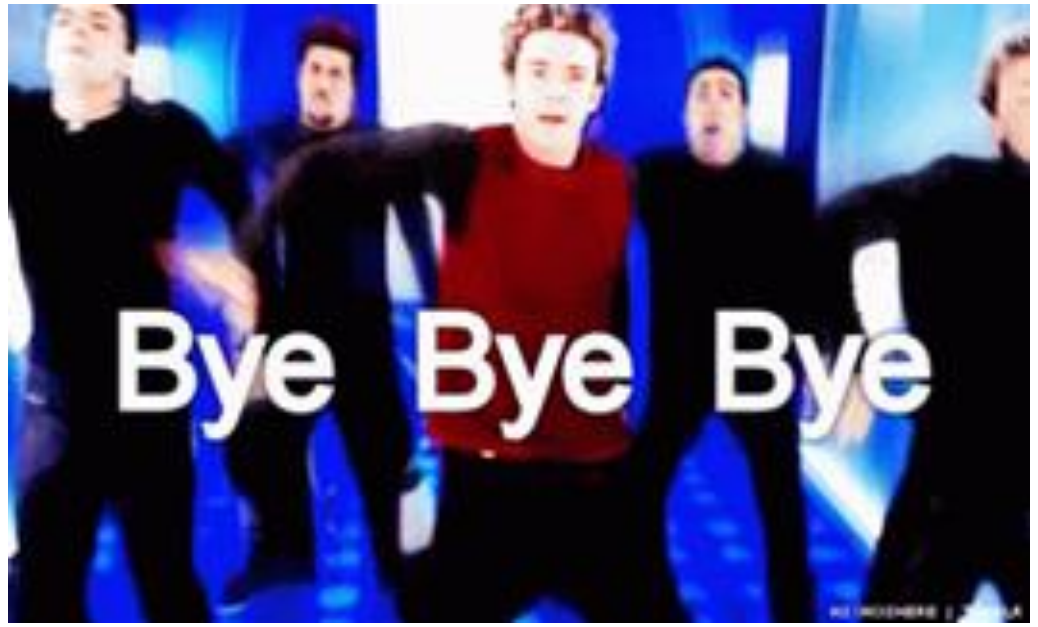

GC-PRO

Final instructions

1. After completing the 2 skills checks in the online form, please press **submit** to record your answers.
    a. We recommend you **download a PDF of your responses** to save for next week's discussion (click on the button at the bottom of the submission page).

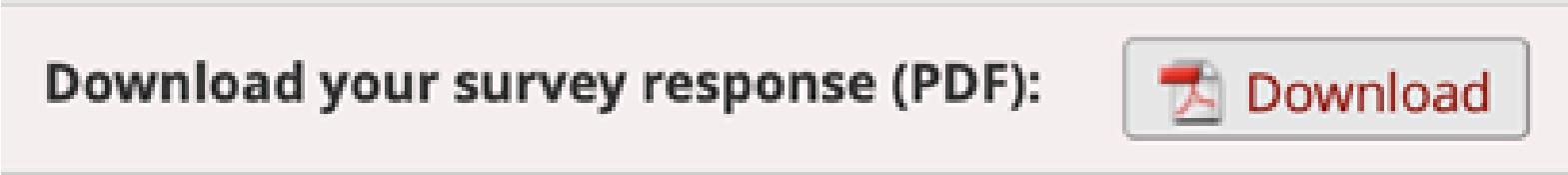

1. **Continue onto the next skill**, "Main Messaging in Education and Action Planning", and complete the corresponding skills checks.

GC-PRO

Thank you!

Heather Zierhut:
zier0034@umn.edu

Debi Cragun:
dcragun@usf.edu

Elena Fisher:
fishe912@umn.edu

References

- (Standardized patient quotes): Manuscript in press at Journal of Genetic Counseling: Cragun et al., "Unveiling genetic counseling skills: Developing an online training course and analyzing counselor communication practices" (2025)
- Unpublished data from the Genetic Counseling Processes Result in Outcomes (GC-PRO) study, stage 1

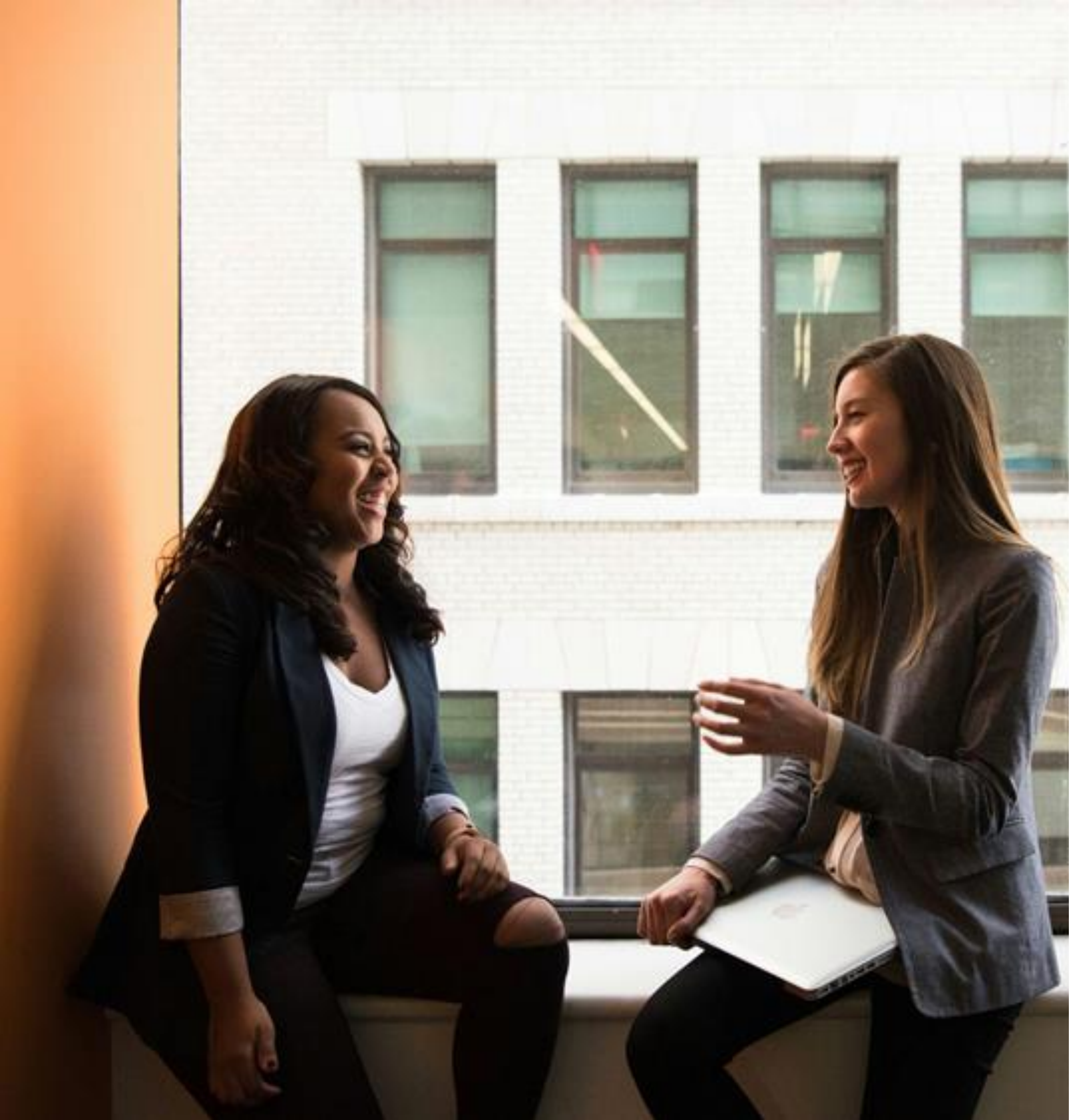

Genetic Counseling Processes Result in Outcomes:

Main Messaging

Skill #3

Week 1

Watch this video to review content together, or move through the slides at your own pace!

Confidentiality reminder:

Please do not share the content of any GC-PRO training materials with anyone, including other participating GC-PRO genetic counselors at your institution. Thank you!

GC-PRO

Outline

1. Skill overview & why main messaging matters
2. Ways to apply main messaging
3. Decide how you will use "Ask-Tell-Ask" in your sessions

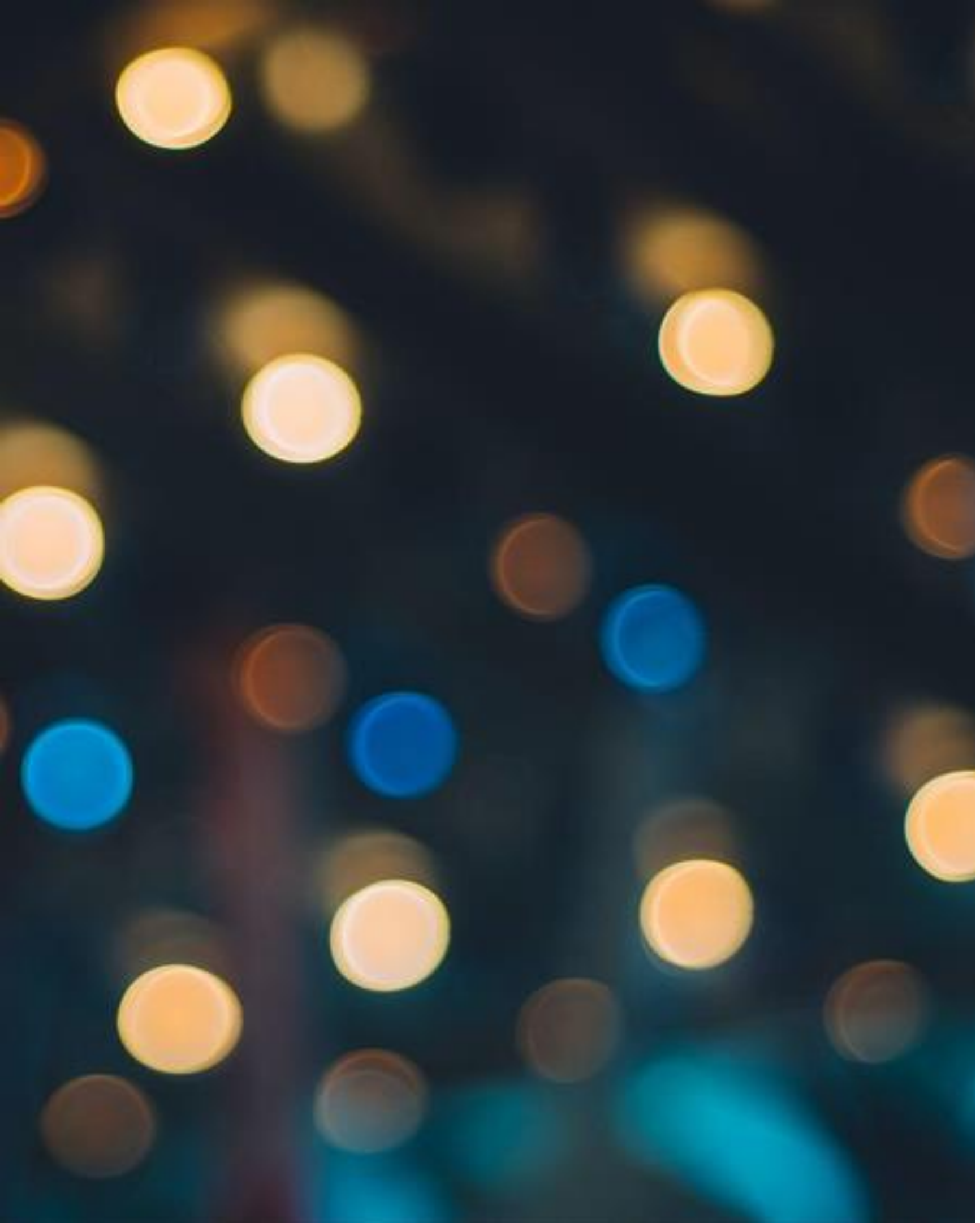

Rationale for use and evidence behind the skill

Main Messaging - Overview

- **First, assess** what they know or think about a topic or option
- Next, give a **concise summary** and focus on **<u>key</u> messages**
    - **Avoid jargon**
    - **Avoid "lecturing"** (i.e., *>20 seconds of continuous talking by GC)*
- **Eliminate information** that is not *explicitly desired by* or *relevant to* the patient
- **Facilitate planning** to support patient activation and avoid patient inaction or confusion after the visit

GC-PRO

Main Messaging - When?

2 areas:

1. **Educating**

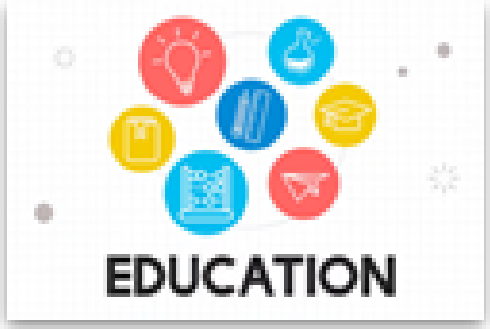

Involves the **main educational & decision making component(s)** of a session (e.g., testing options, medical management, test results)

2. **Action Planning**

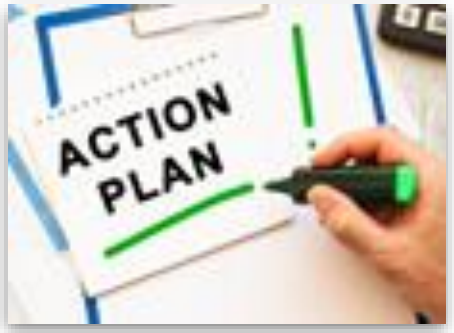

Collaboratively developing a plan which outlines **actions the patient can take** based on their risk assessment, decision made, test results, or management guidelines (usually near end of session)

GC-PRO

Main Messaging

1. Educating

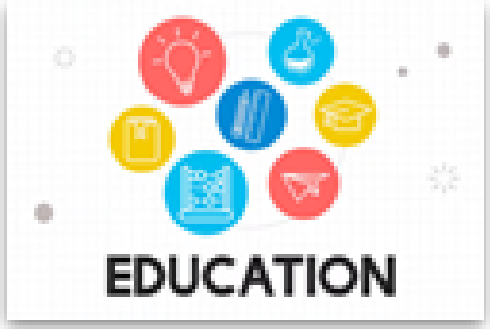

Involves the **main educational & decision making component(s)** of a session (e.g., testing options, medical management, test results)

Why main messaging in EDUCATING & decision making components of the session?

Why use when educating...

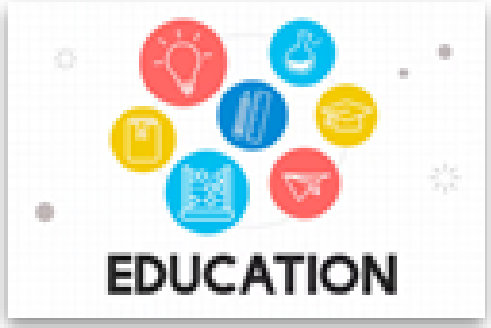

Focusing on the **most important <u>and</u> relevant** points aids in *comprehension* and *engagement*

- Patients prefer when you **remove educational elements** that are too *technical*, too *detailed*, or *not directly related* to their situation (Redlinger-Grosse et al., 2021)
- Jargon and technical explanations, when not asked for, can cause:
  - Information- and numeracy-overload
  - Confusion
  - Disengagement
  - Added time that is not meeting the patient's needs

GC-PRO

Why use when educating…

Allows you to meet each patient's *personal* levels of **interest** and informational **needs**

- Patients have **varying levels of interest** in the information

"[GC] presented a lot of really interesting information, but **most of it didn't really seem relevant**…" - *Standardized patient in lower-rated session*

- **Taking turns talking** and **checking in** is preferred

…**back and forth dialogue** gives you an opportunity to bring up questions organically where it fits in the conversation…**back and forth dialogue** helps also make you feel more comfortable…- *GC-PRO patient interview*

Main Messaging

Why main messaging in ACTION PLANNING components of the session?

2. Action Planning

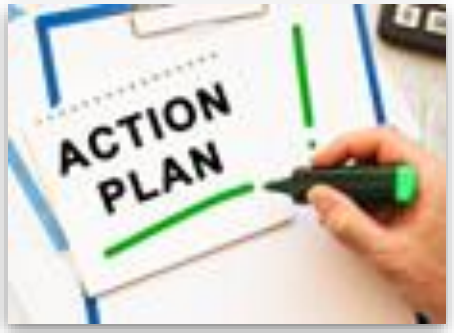

Collaboratively developing a plan which outlines **actions the patient can take** based on their risk assessment, decision made, test results, or management guidelines (usually near end of session)

GC-PRO

Why use when action planning...

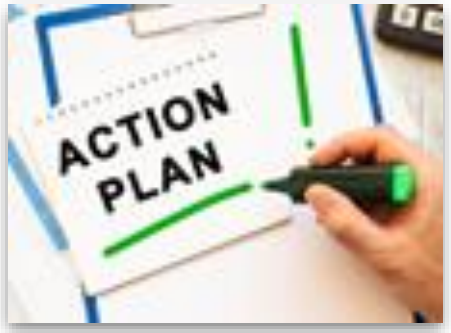

Information **aligned with a purpose and action** improves patients' post-session **anticipatory guidance**

- GCs play a critical role in coordination of care and follow-up

  (Zierhut et al., 2016; *manuscript under review at JoGC)*

- Patients prefer **clear guidelines and information** on next steps

"...a week later, my answer is, "Oh, I wish I would have been **more clear on what to expect** beyond that initial first next step." - *GC-PRO patient interview*

"...being **very clear about timelines** and **what to expect** leaving it... so it just kind of checked off **everything that I would hope for** in a counseling session..." - *Standardized patient in higher-rated session*

GC-PRO

Why use when action planning...

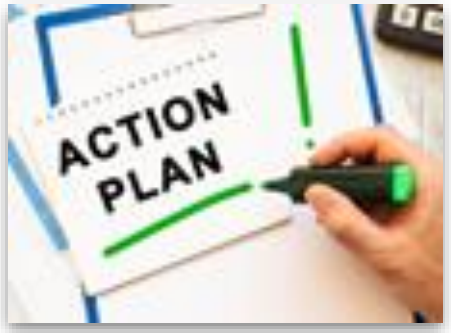

Information **aligned with a purpose and action** improves patients' post-session **anticipatory guidance** *(continued)*

- Patients appreciate *guiding* and *clarifying* questions
- Helping patients **think through a plan** increases their likelihood of **acting on the information**, especially at the end of the session (Winchester et al., 2022)

"[GC] was extremely helpful in asking questions that **helped me clarify my own thoughts**." - *Standardized patient in higher-rated session*

"...helped me **make a plan** rather than *telling me* the plan." - *Standardized patient in higher-rated session*

GC-PRO

Why use main messaging *overall*?

Increases **efficiency**

- **Your time and the patient's time are valuable.** Main messaging can increase efficiency, which is one of the Institute of Medicine's measures of healthcare quality.
- *As a reminder:*
  - From GC-PRO stage 1, patients' perception of the **quality** of the GC session was NOT associated with session time
  - Patients appreciate you being thorough *AND* relevant
    (manuscript under review at JoGC)

GC-PRO

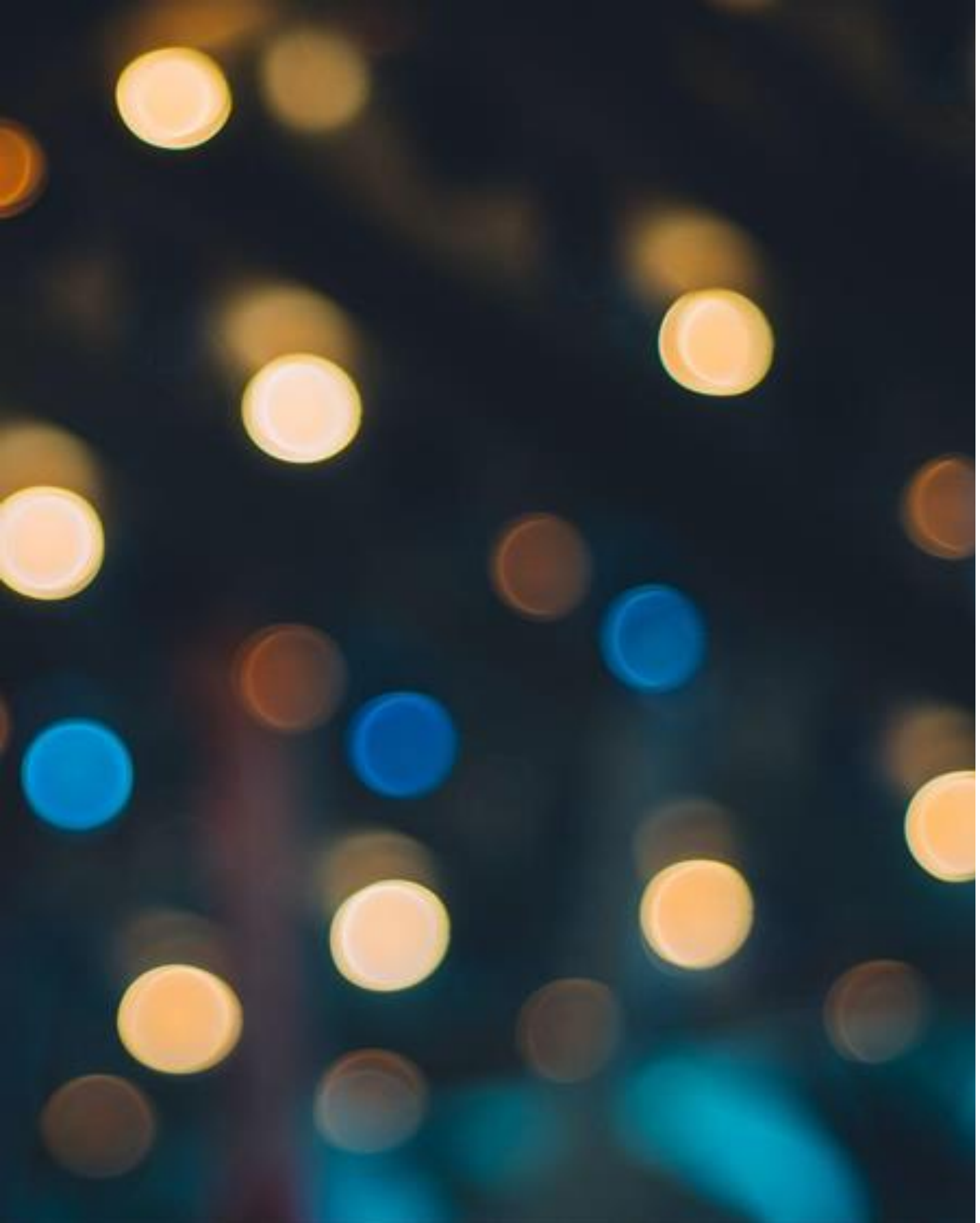

Explanation of main messaging, how to use it, and examples

GC-PRO

Main Messaging - 3 Steps:

For *each* main message:

1. **Ask:**
    - Assess prior knowledge or thoughts with an *open-ended* question
2. **Tell:**
    - Give *brief* amounts of information (1 main message at a time)
3. **Ask:**
    - *Elicit* what they think about the main message and determine whether more details are desired BEFORE moving onto the next main message

GC-PRO

Main Messaging Step 1: *Ask*

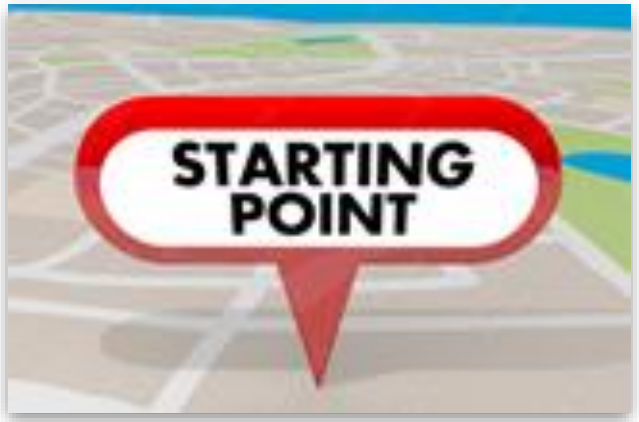

Assess prior understanding with an **open-ended question**

- **BEFORE** providing each main message or informational point, begin with an *open-ended question*
    - Many GCs elicit prior knowledge at the *beginning*, but not ***throughout*** the educational process
    - Some GCs assess with close-ended questions or questions that are either overly broad or not related to the main message
- Assessing BEFORE each point will break up lecturing and help decide if info is **necessary** to provide vs. what is **already known**

Main Messaging Step 2: *Tell*

THE MAIN MESSAGE

Give **brief** amounts of information (1 main point at a time)

- START with **one main message (typically no more than 2-3 sentences at a time)**
    - GCs traditionally provide background details first and *lead up to* the main point(s)
    - Instead, prioritize the "**3 to 5 most important things**" the patient needs to know in order to make a decision or take action after the visit
- **Remove technical info/jargon** and educational elements not *directly related* to the patient's situation

GC-PRO

Main Messaging Step 2: What to *tell*?

Modified transcript from a real GC-PRO session:

"So with the diagnostic testing, they do also ask us to discuss and offer it because it is available to everybody. Many patients will do noninvasive prenatal testing first. But some may choose to directly go to diagnostic testing. It is available for all pregnancies. We used to do the procedures more commonly because we didn't have as good of blood work. But you know, because we have such good screening, they're not done as routinely. They are done if noninvasive prenatal testing comes back high risk, if something's seen on ultrasound that's concerning, you wanted more information, or if there's a known genetic change in the family that you wanted to test the pregnancy for. In the first trimester, the procedure is called the CVS or the chorionic villi sampling. They take tissue from the placenta. They go either transabdominally with a needle or transvaginally with a catheter to collect tissue from the placenta, get the full picture of the placental DNA, and can say yes or no about the different genetic conditions. In the second trimester, there's the amniocentesis. They use ultrasound guidance, collect fluid from the amniotic sac with the needle, the fluid itself would have come off the baby, and it's the first time we can get the full picture of the baby's DNA and say yes or no to different genetic conditions. The invasive procedures go into baby's environment. There are some risks, which do include a risk for a miscarriage, bleeding, cramping, and infections after the procedure. The overall risk is less than 1%, but it's still there. So that's something that we do mention with those procedures."

Main Messaging Step 2: What to *tell*

EDUCATION

How can we "MAIN MESSAGE" this information?

"~~So with the~~ diagnostic testing~~, they do also ask us to discuss and offer it because it is available to everybody. Many patients will do noninvasive prenatal testing first. But some may choose to directly go to diagnostic testing. It~~ is available for all pregnancies~~. We used to do the procedures more commonly because we didn't have as good of blood work. But you know, because we have such good screening, they're not done as routinely. They are done if noninvasive prenatal testing comes back high risk, if something's seen on ultrasound that's concerning, you wanted more information, or if there's a known genetic change in the family that you wanted to test the pregnancy for. In the first trimester, the procedure is called the CVS or the chorionic villi sampling. They take tissue from the placenta. They go either transabdominally with a needle or transvaginally with a catheter to collect tissue from the placenta, get the full picture of the placental DNA, and can say yes or no about the different genetic conditions. In the second trimester, there's the amniocentesis. They use ultrasound guidance, collect fluid from the amniotic sac with the needle, the fluid itself would have come off the baby, and it's the first time~~ we can get the full picture of the baby's DNA and say yes or no to different genetic conditions. ~~The invasive procedures go into baby's environment.~~ There are some risks, which do include a risk for a miscarriage~~, bleeding, cramping, and infections after the procedure. The overall risk is~~ [in] less than 1%~~, but it's still there. So that's something that we do mention with those~~ [of] procedures."

Main Messaging Step 3: *Ask*

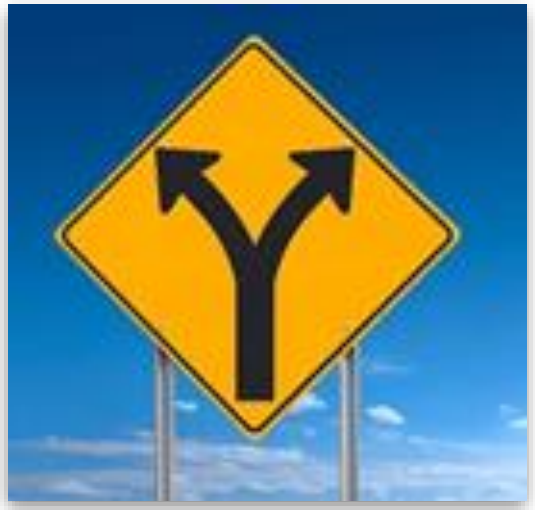

Elicit thoughts, feelings, and/or **desire for more info**

- Ask one *or* both of the question types below:
  - Generalized, ***open-ended*** question (e.g. "What do you think about what I just shared?")
  - ***Targeted*** question (e.g. "Would it be helpful for me to go into more details about [X topic]?")
- ONLY add more information if the patient explicitly desires more
- Then, **move onto the NEXT main message** and apply Ask-Tell-Ask again for that message

Main Messaging Step 3: *Ask*

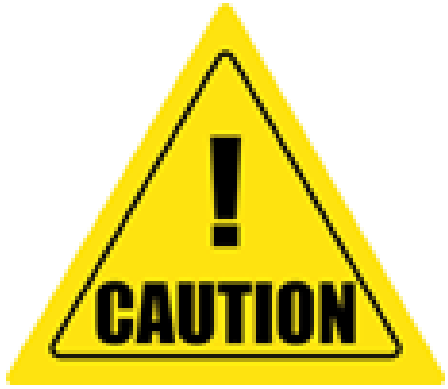

NOTE of caution from GC-PRO part 1

- Asking generalized **closed-ended questions** often **closed down conversation**
- "*Any questions about that?*" usually led patients to say "*no*"
- Patients shut down even more when GCs reinforced a "*no*" response

"great"   *"perfect"*   *"sounds good"*

GC-PRO

Main Messaging - EDUCATING example

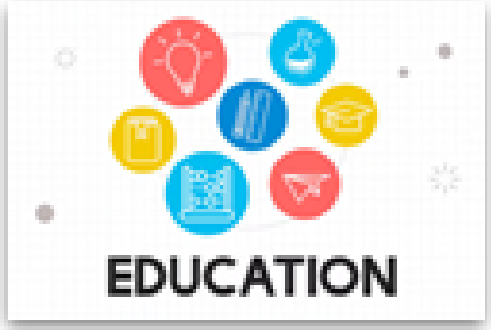

ASK: "Before we move on, what questions do you have about these types of tests?"

Patient: "One thing I don't understand is diagnostic tests. How are those different from the other blood tests you talked about before?

TELL: "Thanks for bringing that up! We use "diagnostic" to refer to tests that give a diagnosis or a "yes or no" answer. Screening refers to tests that can only tell us how high the chance is for the pregnancy to have the condition."

ASK: "What is your reaction to these two types of tests?"

Patient: "I do like getting a yes or no answer, but I'm not sure about the risks you mentioned."

GC-PRO

Main Messaging - DECISION MAKING example

ASK: “Now that we’ve talked about all these tests, what do you think might be the best option for you?”

Patient: “I’m leaning toward [option 1] but it’s hard to know what to do.”

TELL: “That’s helpful to know which option you’re learning toward. Choosing can be hard since there is no one “correct” option. I’ve seen some people choose [option 1] because of ___, while other people decide on [option 2] because ___ is more important to them.”

ASK: “What other reasons might you have for preferring [option 1] instead of [option 2]?”

Patient response: “Well, I also think [option 1] will help me……., but [option 2] will….”

Main messaging: Video example

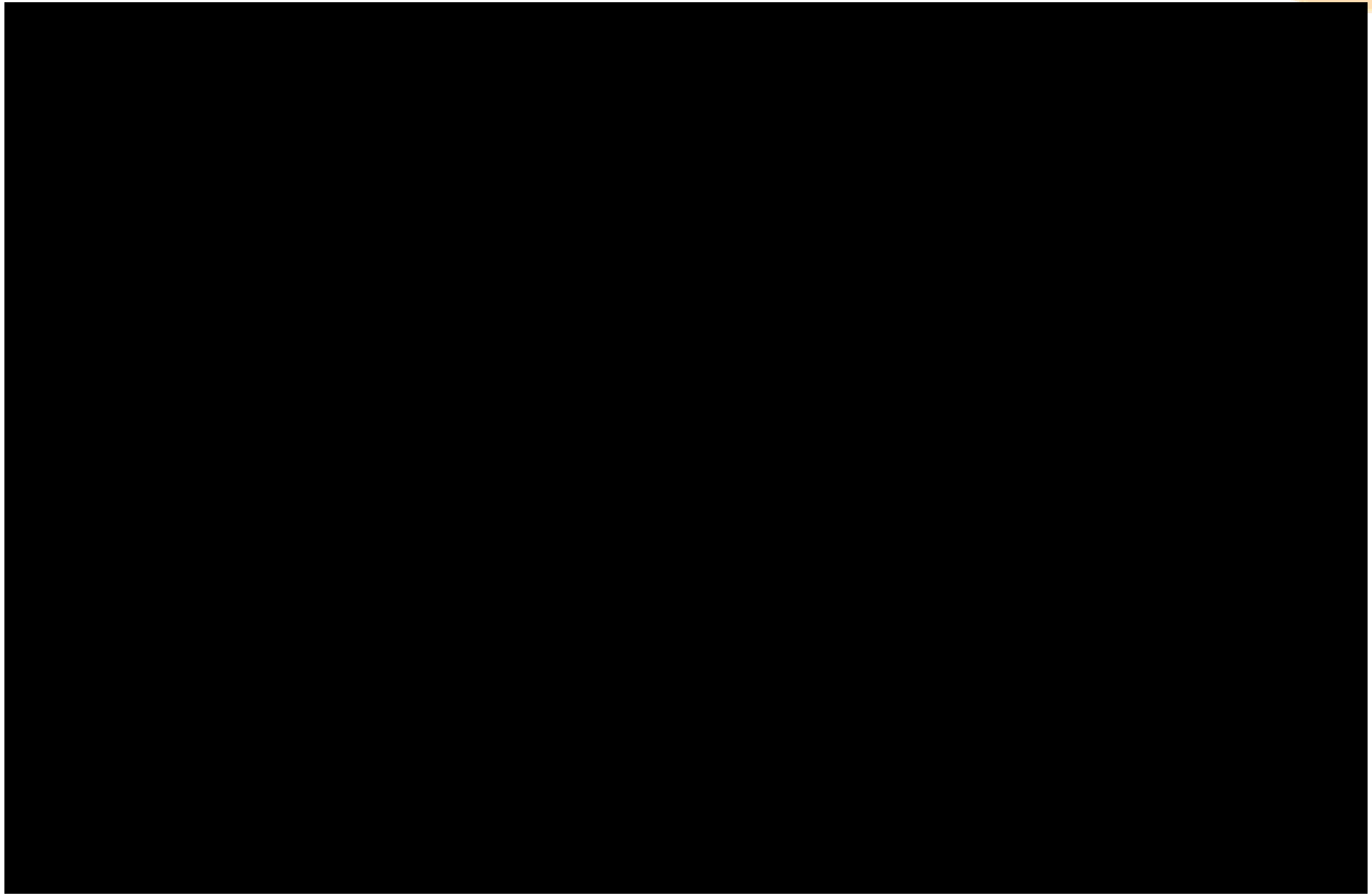

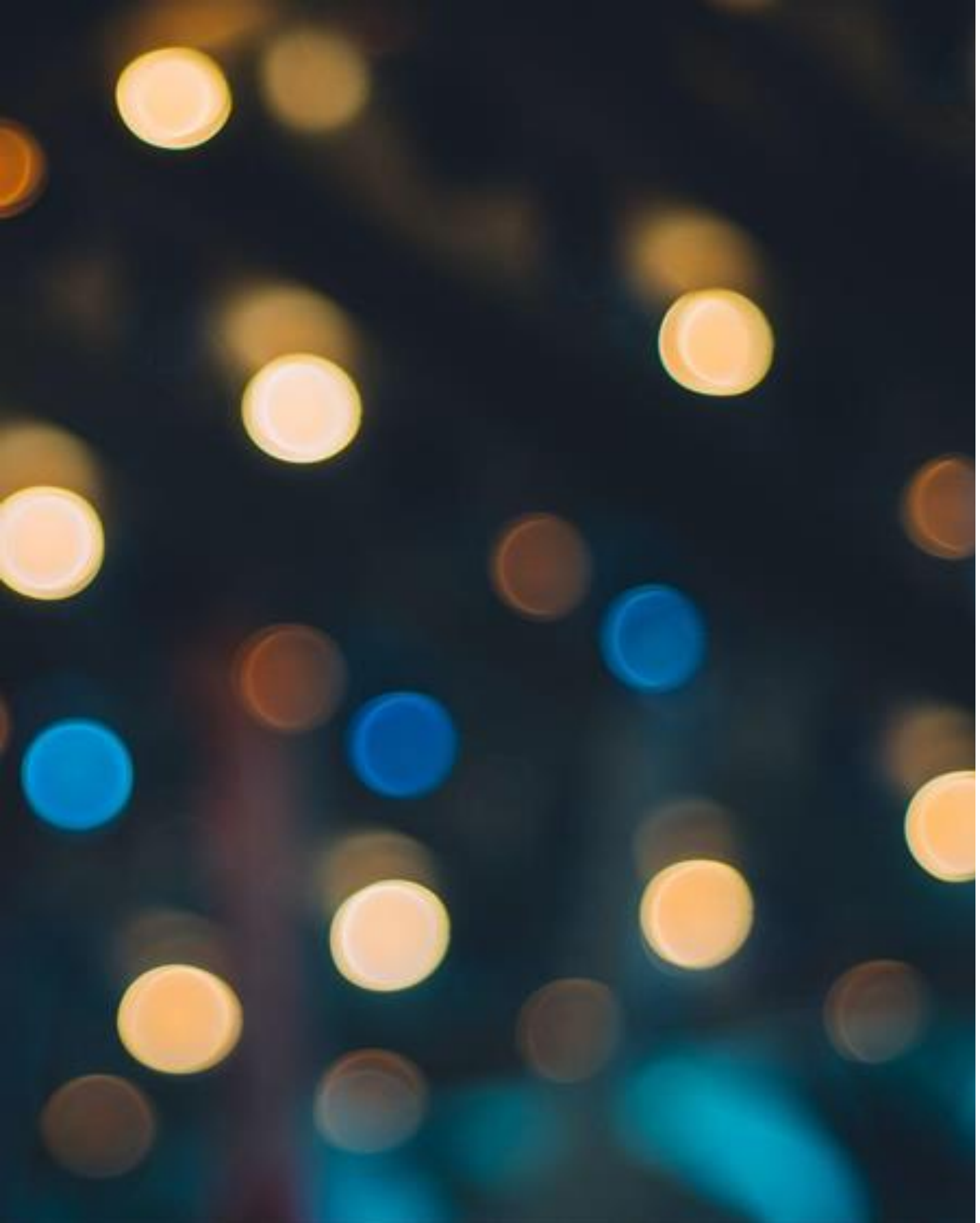

What works for YOU? Decide how to use main messaging in your sessions

GC-PRO

Ask-Tell-Ask

It's as easy as 1, 2, 3...

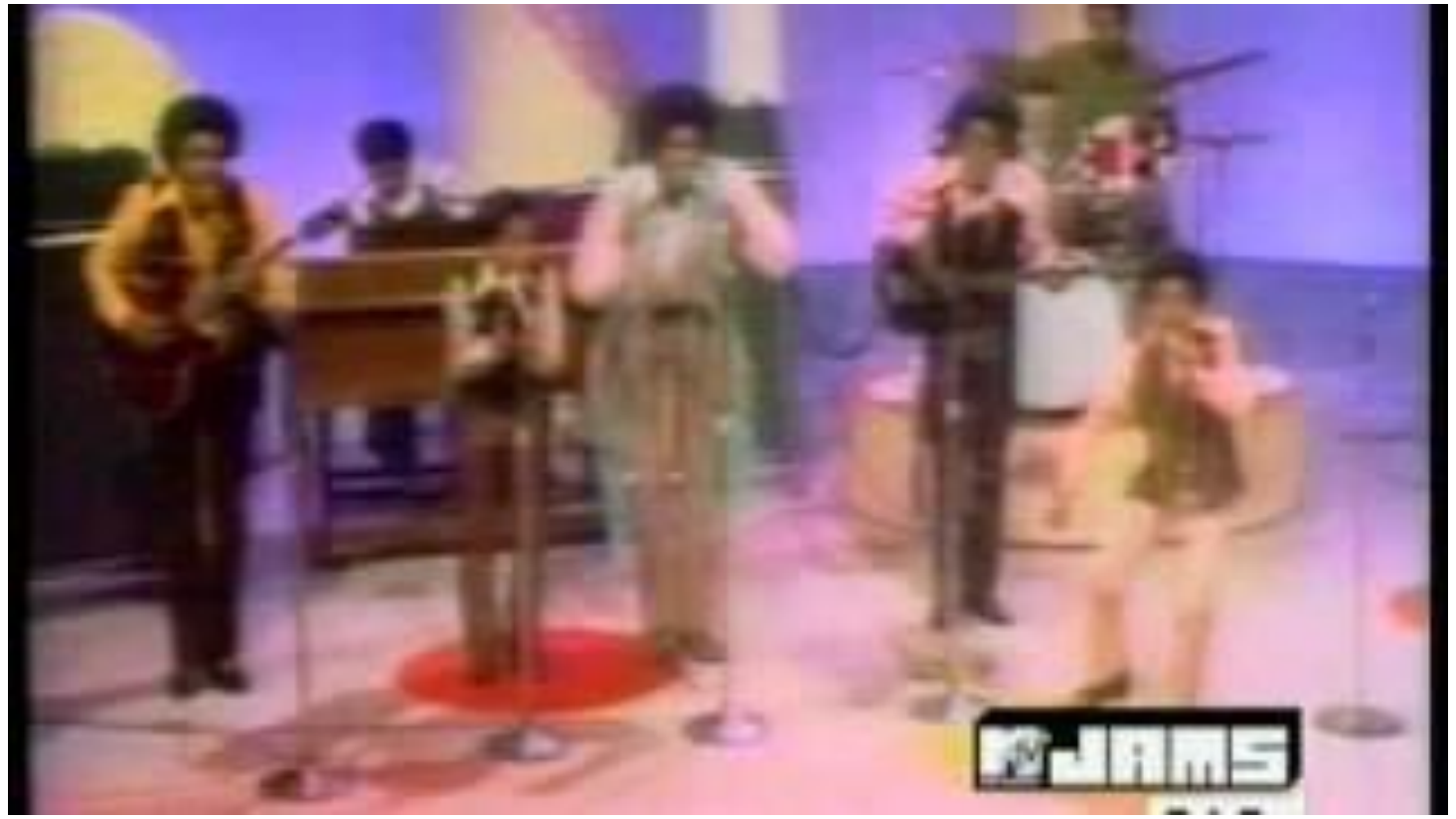

Taking turns *{educating & action planning}*
with you and me!

GC-PRO

Skills Check Time!

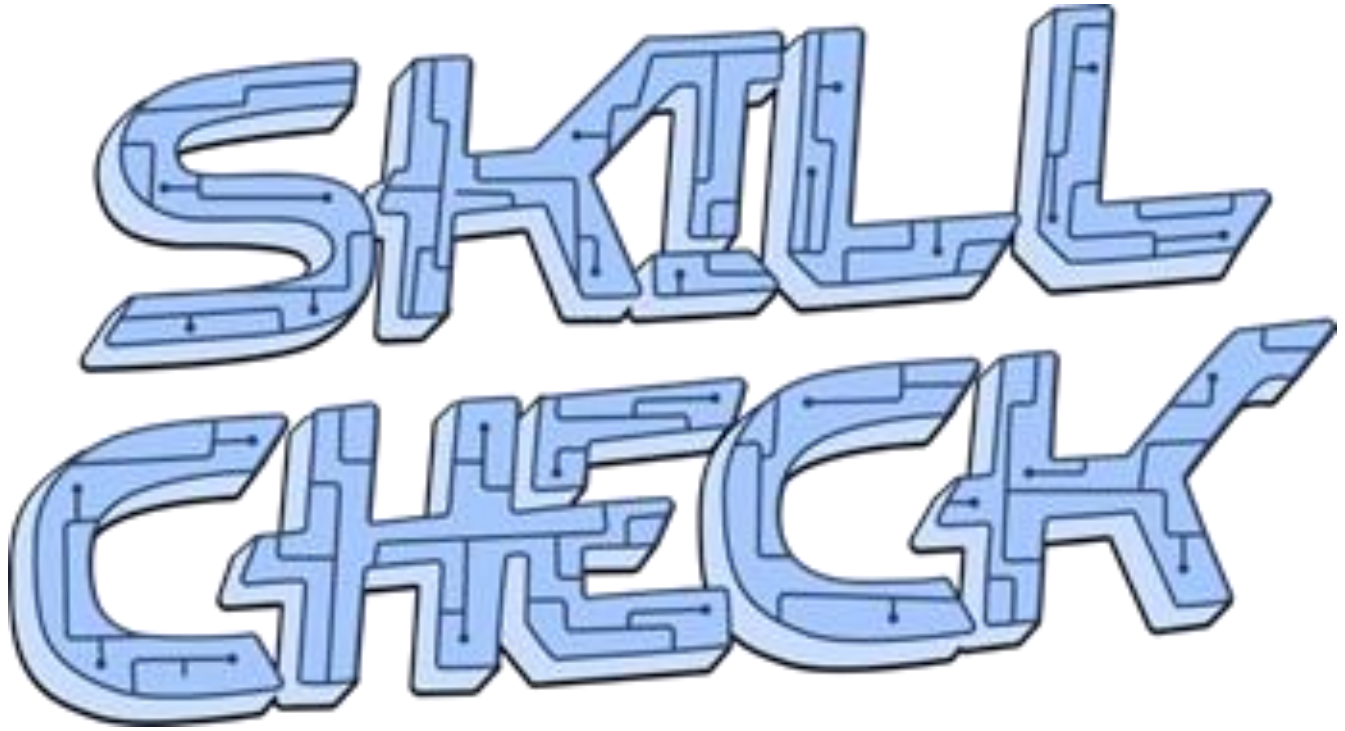

Click on the **“Main Messaging - Skills Check”** personalized link from the GC-PRO Training Introduction email.

An interactive form will open on your browser.

Please follow the instructions in the form while viewing these slides.

Skills Check 1: Finding the main message

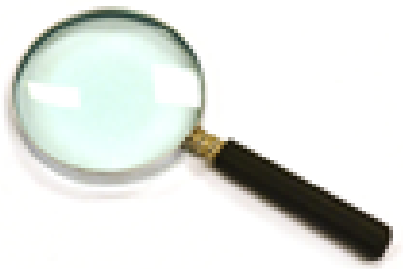

1. An **example transcript** of a GC session from your specialty will be presented. Similarly to "Streamlining Information Gathering," **use the "What + How + Why" criteria** to identify the main messages.

What does the patent *absolutely* need to know in order to make a decision and/or take action for their *primary* indication/concern?

How might knowing this information *change* the patient's medical decision-making or action steps?

Why would knowing this information *help* the patient?

GC-PRO

Skills Check 1: Finding the main message

2. **Sort** the transcript sentences into the following 3 categories of information:

- ✓ Should definitely be **kept** (e.g. the main messages)
- ? **Might be useful** depending on the patient's interest/needs
- ✗ **Unnecessary** information for most patients

GC-PRO

Skills Check 2: Creating YOUR main messages

1. Consider the **top 3 most important points** you feel your patients should "take home" for your *most common* session indication.
    a. (These could be from the Educating section, Action Planning section, or a combination of both)

1. Start by writing out the **1-3 sentence main message** for each of the three points **(TELL)**

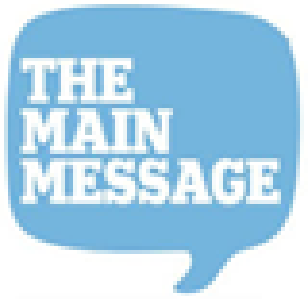

1. Next, list **details** that SOME patients *may* want you to go into for each point

Skills Check 2: Creating YOUR main messages

4. Lastly, write out **2-3 "ASK" questions** per main message that would *help you understand* whether the patient desires or needs more info OR elicits their reactions about the info provided

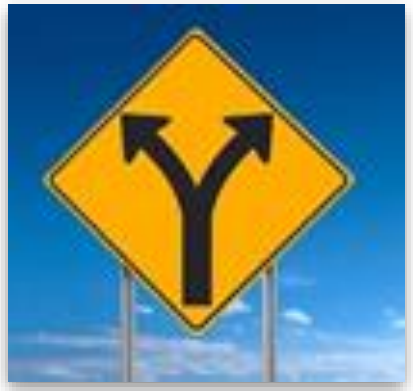

As a reminder, you can use:

→ Generalized, ***open-ended*** questions (such as "What do you think about what I just shared?")

→ ***Targeted*** questions (such as "Would it be helpful for me to go into more details about [topic]?")

GC-PRO

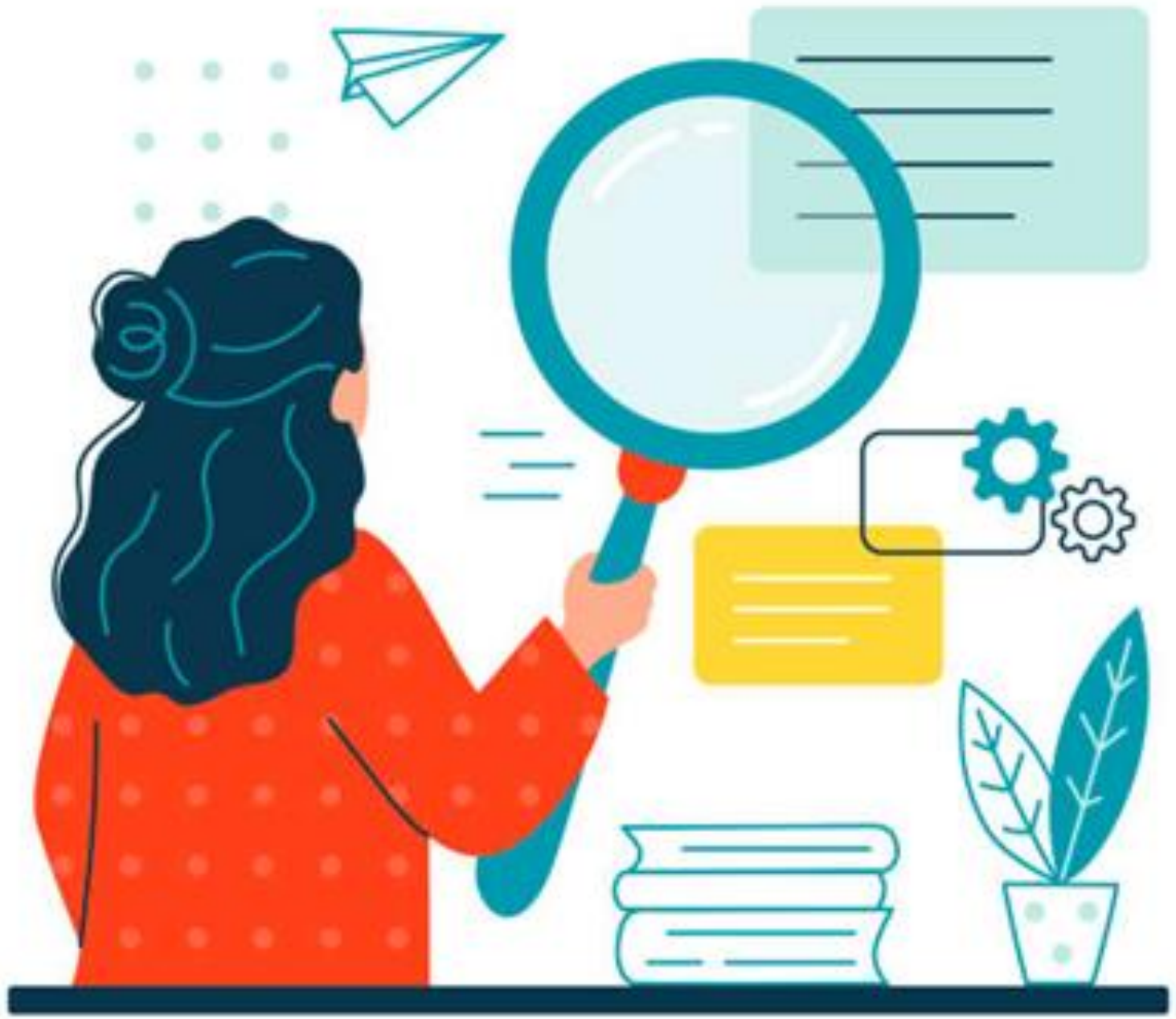

Lastly, how will the researchers be analyzing this skill?

Transparency regarding criteria used to assess this skill

GC-PRO

Criteria for main messaging

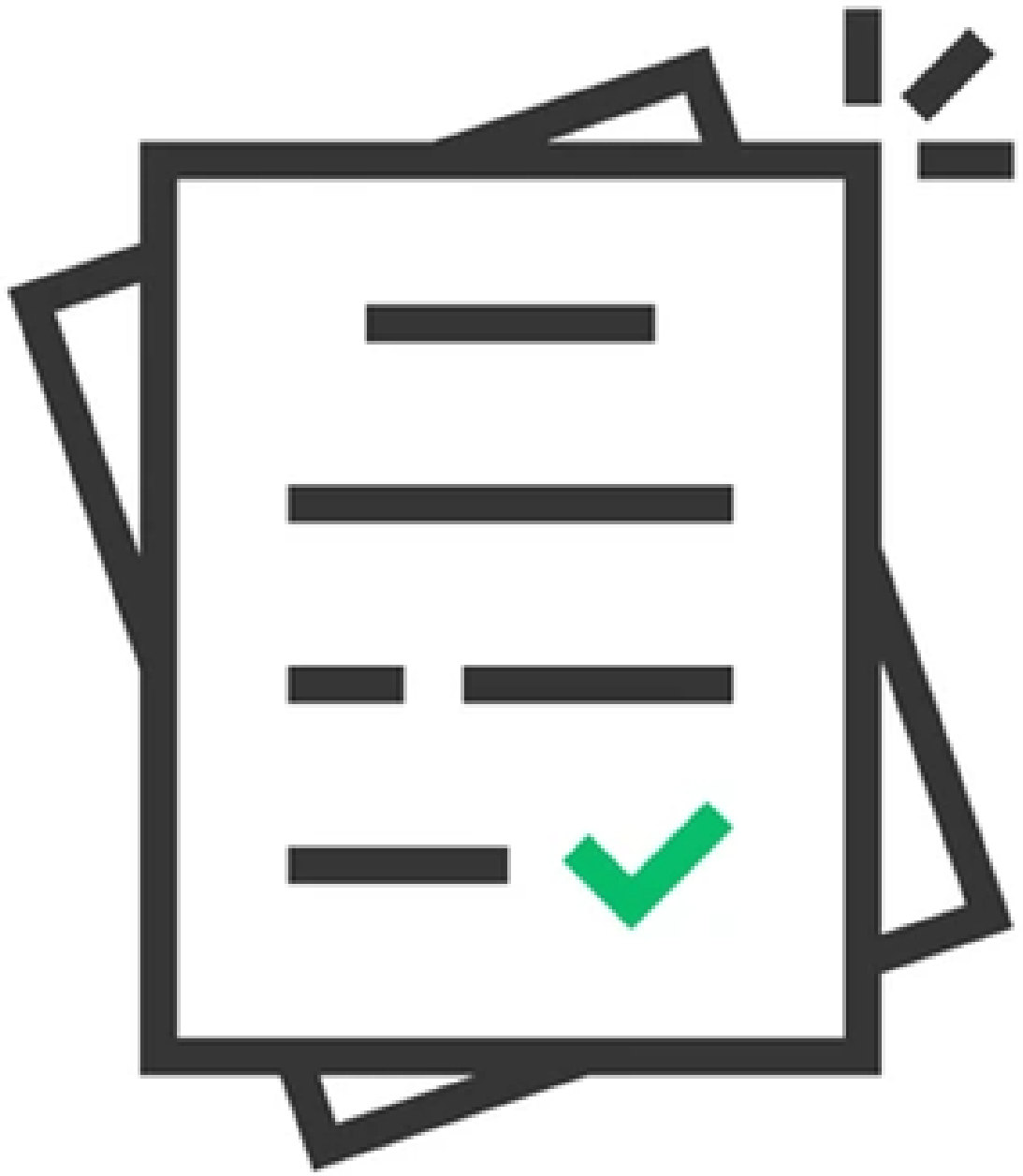

- ❑ **Percentage of time in information-giving** versus other parts of the session
- ❑ Number of segments with **>20 seconds of continuous talking** while educating
- ❑ *Education section:*
    - ❑ **Number of times** Ask-Tell-Ask used
- ❑ *Action planning section:*
    - ❑ **Number of times** Ask-Tell-Ask used

GC-PRO

Main messaging

is as easy as:

1) Ask
2) Tell
3) Ask

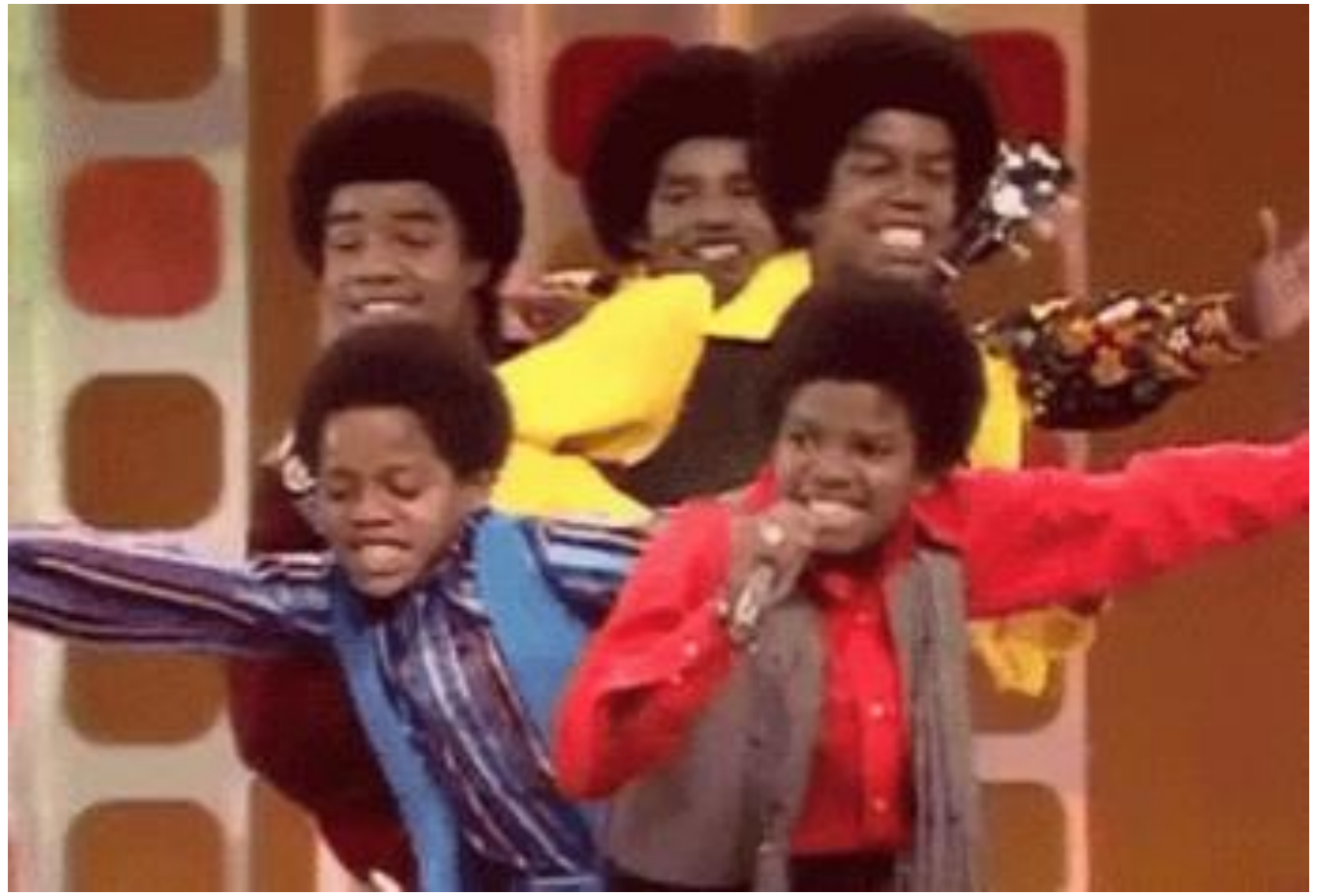

When educating and action planning,
taking turns is as easy as 1, 2, 3!

GC-PRO

Final instructions

1. After completing the 2 skills checks in the online form, please press **submit** to record your answers.
   a. We recommend you **download a PDF of your responses** to save for next week's discussion (click on the button at the bottom of the submission page).

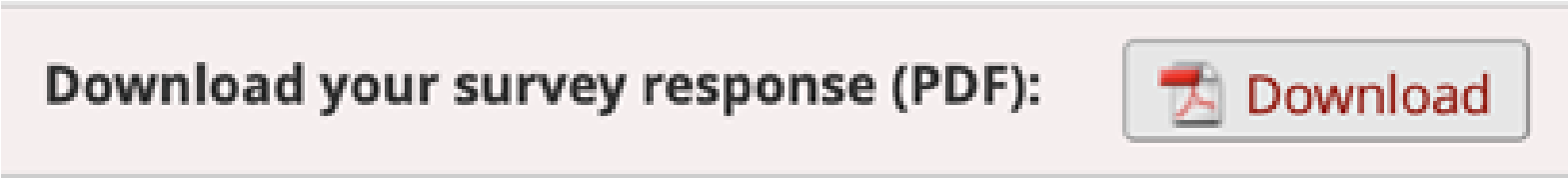

1. If you completed the 3 powerpoints and skills checks in order, **you have now COMPLETED all of your Week 1 activities!**
2. *You do not need to do anything else at this time,* beyond keeping track of your survey responses/questions for next week's group discussion.

GC-PRO

Thank you!

Heather Zierhut:
zier0034@umn.edu

Debi Cragun:
dcragun@usf.edu

Elena Fisher:
fishe912@umn.edu

GC-PRO

GC-PRO

References

In order of appearance:

- Redlinger-Grosse, K., MacFarlane, I. M., Cragun, D., & Zierhut, H. (2021). A Delphi study to prioritize genetic counseling outcomes: What matters most. Journal of Genetic Counseling, 30(3), 676-692.
- (Standardized patient quotes): Manuscript in press at Journal of Genetic Counseling: Cragun et al., "Unveiling genetic counseling skills: Developing an online training course and analyzing counselor communication practices" (2025)
- Zierhut, H. A., Shannon, K. M., Cragun, D. L., & Cohen, S. A. (2016). Elucidating genetic counseling outcomes from the perspective of genetic counselors. Journal of Genetic Counseling, 25, 993-1001.
- Winchester, B., Cragun, D., Redlinger-Grosse, K., Walters, S. T., Ash, E., Baldry, E., & Zierhut, H. (2022). Application of motivational interviewing strategies with the extended parallel process model to improve risk communication for parents of children with familial hypercholesterolemia. Journal of Genetic Counseling, 31(4), 847-859.
- Agency for Healthcare Research and Quality: Six Domains of Healthcare Quality - https://www.ahrq.gov/talkingquality/measures/six-domains.html#_ftn1
- Unpublished data from the Genetic Counseling Processes Result in Outcomes (GC-PRO) study, stage 1

GC-PRO
